# Classic Selective Sweeps Revealed by Massive Sequencing in Cattle

**DOI:** 10.1371/journal.pgen.1004148

**Published:** 2014-02-27

**Authors:** Saber Qanbari, Hubert Pausch, Sandra Jansen, Mehmet Somel, Tim M. Strom, Ruedi Fries, Rasmus Nielsen, Henner Simianer

**Affiliations:** 1 Animal Breeding and Genetics Group, Department of Animal Sciences, Georg-August University, Goettingen, Germany; 2 Chair of Animal Breeding, Technische Universitaet Muenchen, Munich, Germany; 3 Departments of Integrative Biology and Statistics, University of California at Berkeley, Berkeley, California, United States of America; 4 Institute of Human Genetics, Helmholtz Zentrum München, Munich, Germany; Stanford University, United States of America

## Abstract

Human driven selection during domestication and subsequent breed formation has likely left detectable signatures within the genome of modern cattle. The elucidation of these signatures of selection is of interest from the perspective of evolutionary biology, and for identifying domestication-related genes that ultimately may help to further genetically improve this economically important animal. To this end, we employed a panel of more than 15 million autosomal SNPs identified from re-sequencing of 43 Fleckvieh animals. We mainly applied two somewhat complementary statistics, the integrated Haplotype Homozygosity Score (iHS) reflecting primarily ongoing selection, and the Composite of Likelihood Ratio (CLR) having the most power to detect completed selection after fixation of the advantageous allele. We find 106 candidate selection regions, many of which are harboring genes related to phenotypes relevant in domestication, such as coat coloring pattern, neurobehavioral functioning and sensory perception including KIT, MITF, MC1R, NRG4, Erbb4, TMEM132D and TAS2R16, among others. To further investigate the relationship between genes with signatures of selection and genes identified in QTL mapping studies, we use a sample of 3062 animals to perform four genome-wide association analyses using appearance traits, body size and somatic cell count. We show that regions associated with coat coloring significantly (P<0.0001) overlap with the candidate selection regions, suggesting that the selection signals we identify are associated with traits known to be affected by selection during domestication. Results also provide further evidence regarding the complexity of the genetics underlying coat coloring in cattle. This study illustrates the potential of population genetic approaches for identifying genomic regions affecting domestication-related phenotypes and further helps to identify specific regions targeted by selection during speciation, domestication and breed formation of cattle. We also show that Linkage Disequilibrium (LD) decays in cattle at a much faster rate than previously thought.

## Author Summary

Domestication of cattle had a major impact on human civilization by providing protein and physical power for agrarian life style. Domestication followed by breed formation has likely left detectable signatures within the genome of modern cattle. Current cattle breeds, for instance, have a more uniform appearance and milder temper than their wild ancestors. The elucidation of these signatures of selection is of interest to identify domestication-related genes that help to genetically improve this economically important species. The development of novel sequencing technologies has enabled higher-resolution genomic analyses of past selection. In this paper, we exploited whole genome sequencing along with multiple statistical metrics to identify regions/genes putatively targeted by selection. We show strong signals of selection near to several candidate genes related to domesticated phenotypes such as coat coloring, neurobehavioral functioning and sensory perception, including KIT, MITF, MC1R, NRG4, Erbb4, TMEM132D and TAS2R16. By means of association mapping we additionally show that candidate selection regions for appearance traits overlap with major coat color QTLs. Our study demonstrates the utility of population based techniques for detecting past selection and is the first attempt to localize signatures of past selection in cattle based on massive re-sequencing of the entire genome.

## Introduction

The available genetic and archaeological evidences date cattle domestication back to the Neolithic period, around 10,000 BCE [1], [2]. Modern cattle are thought to have originated from multiple independent domestication events of aurochs (B. primigenius) primarily in southwest Asia and south Asia, resulting in the humpless taurine (B. taurus) and the humped zebu (B. indicus) groups respectively [3], [4]. Domestication of cattle had a major impact on human civilization as they provided physical power in agriculture and were a major source of milk, meat and leather products.

Domestication of cattle provides an excellent model of animal evolution. During the domestication process, cattle have adapted in morphology, physiology and behavior to captive life, and have been subject to artificial selection imposed by humans to increase yield, fertility and other processes. As a result, more than 900 breeds, each with distinct characteristics, have emerged throughout the world [5]. The phenotypes associated with domestication include milk and meat production, fertility, appearance including coat coloration, decreased fearfulness, social motivation, and mild temper [6]. The selection affecting these phenotypes has left detectable signatures of selection within the genome of modern cattle [7].

The signatures of selection in the genome, as a beneficial mutation arises and rapidly increases in frequency in the population, can be detected as (i) reduced local variability, (ii) deviations in the Site Frequency Spectrum (SFS) and (iii) increased linkage disequilibrium and extended haplotype structure. These signatures can be used to screen a genome for genes involved in recent adaptation. Numerous statistics have been developed aiming at detecting selection [8], [9], [10], [11], .

Previous genome-wide studies to detect positive selection in cattle have used SNP arrays, which suffer from ascertainment biases caused by the process used to discover SNPs [14], [15], [16] and limited resolution [17], [18], [19], [20], . In addition, these studies have focused on a single selection signature statistic that typically only detects selection during a certain time in the past. Many selection signals may, therefore, have remained un-detected by previous studies.

In this study, we use whole-genome re-sequencing data of 43 Fleckvieh animals [23], a German dual purpose cattle breed. We apply two different statistics, the integrated Haplotype Homozygosity Score (iHS) [12] and the Composite of Likelihood Ratio (CLR) [11] to detect past selection. iHS finds maximal power when a selected allele segregates at intermediate frequencies in the population, whereas the CLR statistics has most power right after the selected allele has gone to fixation. For this reason, the two statistics are complementary in the type of selection that they detect. Using whole genome sequence information rather than genotypes for pre-selected SNP panels avoids the problems caused by ascertainment. This design thus, provides additional power to detect selection missed by previous studies. In addition, we conduct a Genome-Wide Association mapping Study (GWAS) on appearance traits. We find evidence of strong signatures of selection in cattle during speciation, domestication and breed formation exemplified by several striking selective sweeps co-localized with major QTLs.

We also use the direct sequencing data to examine the pattern of Linkage Disequilibrium (LD) in the Fleckvieh breed. A detailed profile of LD over the entire genome is a quantity of interest, especially for the use in breeding programs implementing genomic selection. Previous studies of LD structure in cattle populations have used low resolution panels of ascertained SNPs mainly selected based on their minor allele frequency (MAF) and position on the genome [24], [25, among others].

## Results and Discussion

### Allele frequency distribution and LD

The data from Jansen et al [23] analyzed in this study includes roughly 20 times more SNPs than the 700K array previously used in cattle for examining LD. The increased SNP density provides a greater coverage of rare and low frequency SNPs than in any previous study based on SNP chip data.

**Figure 1:**
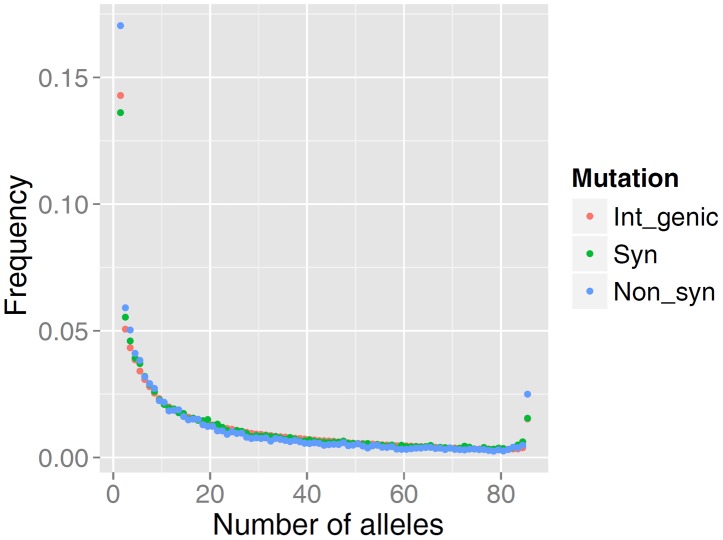
Comparison of the site frequency spectra from resequencing of 43 German Fleckvieh animals. SFS is represented for non-synonymous (Non_syn), synonymous (Syn) and inter-genic polymorphic (Int_genic) variants.

The distribution of allele frequencies follow the same pattern as that observed for high-quality data in many other organisms including human populations [26], [27]. As predicted by population genetics theory, the frequency spectrum is a decreasing function, and among rare alleles there is a slight excess in the proportion of non-synonymous mutations relative to intergenic or synonymous mutations (Figure 1). The relative excess of non-synonymous mutations among rare alleles is presumed to be caused by selection acting on slightly deleterious mutations [26], [27].

**Figure 2:**
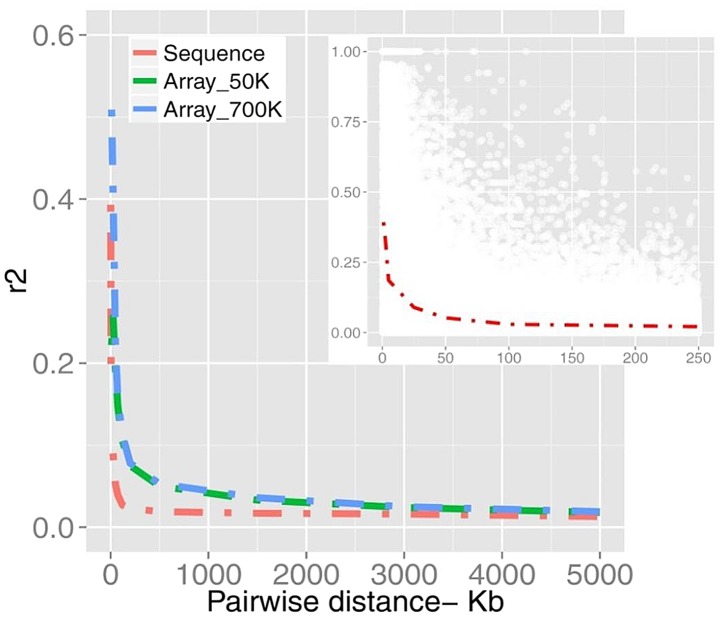
A schematic representation of LD plotted as a function of distance. The decay of LD estimated from bovine SNP arrays of 50K and 700K (n = 1,293) are compared with the sequence data (n = 43). The inner plot displays a higher resolution of LD in pair-wise distances of <250 Kb from sequence data of which *r^2^* values are down-sampled from all pairwise estimates (for more details see Material and Methods).

We found a mean value of *r^2^* = 0.25 (sample SD = 0.29) for SNPs less than 20 kb apart (Figure 2). Table S1 summarizes more properties of LD as a function of physical distance. It is evident that average LD does not extend beyond the inter-marker space of 100 Kb across the genome. Previous studies in cattle however, found strong LD extending over several Megabasepairs [24], [25, among others]. However, LD as measured by *r* ^2^ depends on allele frequencies [28], [25]; and the difference between this study and previous studies may partially be explained by the biased SNPs selection on the Illumina Bovine arrays, where SNPs mainly were ascertained based on allele frequency and a uniform distribution over the genome. Additionally, differences in the sample composition may explain the results, as LD is strongly affected by population structure. Characterizing LD using structured populations leads to an inflation of the LD statistics, which might have affected previous studies. Finally, genotyping error reduces apparent LD, and is a major concern for low- and intermediate-depth coverage re-sequencing data.

To test the degree to which the differences in the LD curves are caused by these factors we examined LD in different scenarios. First we plotted the LD curve after excluding low-frequency variants from sequence data. As shown in Figure S1A, LD persists at a higher level when being estimated with frequent alleles. Second, we sub-selected the sequence data for those SNPs present in the 700k chip and compared LD from this sub-set with sequence and array based LD (Figure S1B). In the new dataset LD decayed faster than original array-based LD possibly due to the different sample composition. However, LD persisted at higher levels compared to the sequence LD due to the different allelic profile (p<0.001). Table S2 summarizes testing the strength of LD in two datasets estimated from SNP pairs in inter-marker distance bins up to 500 Kb. Further, to get insight into the quality of genotypes the concordance rate between sequences vs. array-derived genotypes was evaluated. We observed a significant concordance of 96.9% (±3.4%) based on 38,246 SNPs tested on chromosome 1. The LD curves before and after filtering out all but the highest quality SNPs were overlapped due to the fairly low discordance rate (data not shown). These results demonstrate that LD in cattle decays at a rate much faster than previously thought.

LD-based estimations of past effective population size [7], [25] should be revisited in light of the finding that the ‘true’ sequence-based LD profile is poorly estimated by SNP-chip based LD-estimates. Considering the relatively small effective population size in cattle, population level of LD is unexpectedly low, which suggests that effective population size was considerably larger in the very recent past.

### Localizing selective sweeps

**Figure 3:**
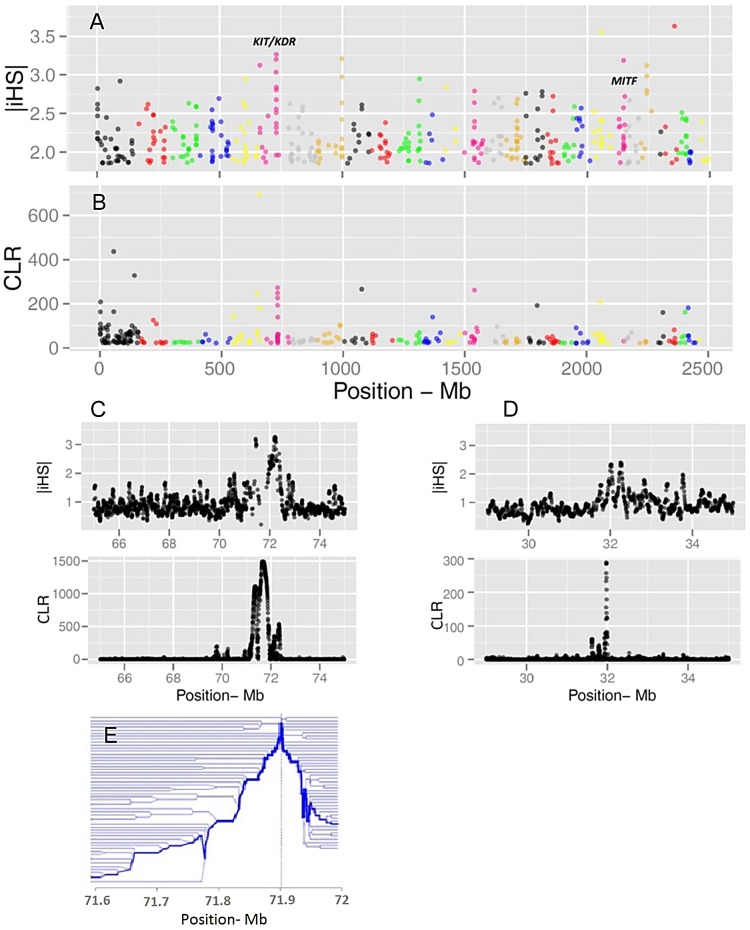
Genome-wide visualization of selection candidates (top 1% signals) localized by |iHS| (A) and CLR (B) metrics. Each dot represents a non-overlapping window of 40 Kb along BTA1 to BTA29. Panels C and D show a high resolution illustration of the candidate regions for the *KIT/KDR* and *MITF* genes, respectively on BTA6 and BTA22. |iHS| is plotted in overlapping windows of 40 Kb in steps of 5 Kb, and a grid size of 5 Kb was chosen for the CLR statistic. Finally, Panel E is a haplotype bifurcation plot of the *KIT/KDR* genes.

Evidence of positive selection was investigated through multiple statistics designed to detect signatures of selective sweeps. We calculated iHS per site and averaged them in non-overlapping 40 Kb windows across the genome, resulting in a total of 62,196 windows (Figure 3). The CLR was estimated using an identical grid size across the genome. We focused the analyses on windows for which the values of the statistics fell in the 99th percentile. Respectively, 68 and 73 candidate regions were identified from iHS and CLR analyses (Tables S3 and S4). There is a substantial overlap between the list of genes identified by CLR and iHS, reflecting the fact that the two tests take advantage of different but correlated patterns of a selective sweep. However, there are also regions solely identified by either metric, possibly because these statistics identify selection acting at different time scales. Two examples of candidate genes are shown in Figure 3: *KIT* and *MITF*, two pigmentation genes on Bos taurus (BTA) chromosomes 6 and 22.

### Putatively selected genes

Tables S3 and S4 summarize statistics for the genomic regions harboring the strongest selection signals. We used DAVID [29] to perform a functional analysis based on the list of all genes in the regions showing signatures of a selective sweep. We found no overall significant enrichment of any particular biological process after correction for multiple testing (data not shown). Nevertheless, we note that genes associated with a number of processes previously implicated in domestication-related changes are present within these regions. These include pigmentation, sensory perceptions, brain and neural system (for review see [6]) along with genes of immunity and blood clotting systems. For clarity and based on a priori interest, we divided genes into functional groups in line with domestication-related changes and discuss each group under separate heading. However, as most genes have pleiotropic effects, selection may possibly act on other functional effects of the genes than those highlighted here. In the following sections, we highlight some results from these analyses.

#### Patterned pigmentation

In mammals, coat color loci influence the development, differentiation, proliferation, and migration of melanocytes, the construction and transport of melanosomes, as well as the synthesis of melanin. In the genome-wide screen, the window with the strongest signal (P_iHS_ = 0.00029, P_CLR_ = 0.00011) coincides with a cluster of tyrosine kinase receptor genes (*PDGFRA, KIT and KDR*) on BTA6 (Figure 3). Among them, *KIT* is a widely studied gene with an important role in several critical pathways including melanogenesis. Genetic variation in the *KIT* gene has been shown to affect coat coloring pattern in a variety of mammals including cattle [30], horses [31], pigs [32] and mice [33]. Another strong selection candidate included microphthalmia-associated transcription factor (*MITF*, P_iHS_ = 0.00172 and P_CLR_ = 0.00709) a major candidate for patterned pigmentation in BTA22 [30]. *KIT* and *MITF* show complex interactions in that *MITF* is needed for the maintenance of *KIT* expression in melanoblasts and *KIT* signaling modulates *MITF* activity and stability in melanocyte cell lines. The mutual interaction between *KIT* and *MITF* is particularly interesting since mutations in any one of them lead to a strikingly overlapping phenotype of early loss of the melanocyte lineage [34].

Another particularly interesting selective sweep candidate in this group overlaps melanocortin 1 receptor (*MC1R*, P_iHS_ = 0.00785 and P_CLR_ = 0.00156) on BTA18 (also see Figure S2A), whose permanent activation results in black coat color, whereas loss of function mutations cause red coat color in different mammals including cattle [35]. *MC1R* gene expression is regulated by the *MITF* and has an autosomal recessive mode of inheritance (also see GWAS section).

Among the top selection candidates, we noticed three genes of the *NRG– Erbb4* signaling pathway. This pathway is involved in the development and progression of melanocytes [36]. Our results revealed typical hitch-hiked patterns for *NRG4* (P_iHS_ = 0.00368, P_CLR_ = 0.00221) and *Erbb4* (P_iHS_ = 0.00203, P_CLR_ = 0.00855) genes along with an extremely deviated SFS for the pro-*NRG2* like locus (P_iHS_ = 0.66899, P_CLR_ = 0.00589)(see also Figures S3 and S4). Interestingly, the same pathway appears to have been targeted by positive selection in humans [37]. Since precursors to pigment cells are also precursors to nerve cells, variants of genes in this pathway are also reported to be associated with various psychiatric phenotypes [38], [39]. Another strong signal that we speculate could be related to coat coloring is the *ULBP3* gene (P_iHS_ = 0.00691, P_CLR_ = 0.00917) shown to be associated with the “sudden whitening of the hair” phenomenon [40].

#### Brain development and neurobehavioral functioning

Domesticated species differ from their wild ancestors, notably in behavioral traits such as reduced fear of humans and aggressiveness [6]. We noticed strong signals standing by some genes underlying extreme neurobehavioral phenotypes and psychiatric disorders (for instance see Figures S5, S6 and S7). Among the most pronounced candidates in our list are neuronal genes *TMEM132D* (P_iHS_ = 0.00087, P_CLR_ = 0.00021), CACNA1C (P_iHS_ = 0.48366, P_CLR_ = 0.00013), *NRXN1* (P_iHS_ = 0.00396, P_CLR_ = 0.00018) and NPAS3 (P_iHS_ = 0.34897, P_CLR_ = 0.00137) reported as candidate QTLs for anxiety-related behavior, major depression and high risk of developing schizophrenia [41], [42], [43], [44]. One interesting observation was the presence of *GRIK3* (P_iHS_ = 0.00076, P_CLR_ = 0.00761) among the genes with the strongest signal of selection. *GRIK3* is a member of glutamine receptors suggested as QTL for reward-related learning [45]. Other putative selection candidate include *OLIG1* (P_iHS_ = 0.00031, P_CLR_ = 0.00016), *LAMC3* (P_iHS_ = 0.00222, P_CLR_ = 0.00325) and *ATL1* (P_iHS_ = 0.00227, P_CLR_ = 0.00557) genes, which all have central roles in the development of brain cortex and formation of axons. Mutations in the *LAMC3* are suggested to cause malformations of occipital cortical development in humans [46]. We speculate that these genes could have been affected by selection targeting at behavioral traits such as a modest temperament during domestication.

#### Sensory perception

We observed signals for selection targeting several sensory functions including olfaction and taste. The putative sweeps on BTA7 and BTA29 contain four clusters of olfactory receptor (OR) family genes (see Table 1). Olfactory receptors detect and identify a wide range of odors and chemosensory stimuli, a necessity to find food, detect mates and offspring, recognize territories and avoid danger (for review see [47]). OR genes are shown to have been under selection in humans [48] and domesticated animals including dog [49], swine [50] and cattle [7]. They are also reported to be duplicated within the bovine genome [51] suggesting that they may be under strong selection for newly evolving functions.

**Table 1:** A partial list of candidate regions revealed by both iHS and CLR analyses.

#	Chr	Gene^1^	Position(bp)^2^	*P*_|iHS|_	*P*_CLR_	Function/association	Reference
1	1	*OLIG1*	1,915,043	0.00031	0.00016	Developing oligodendrocytes	
2	1	*DSCAM*	142,135,568	0.00281	0.00005	Down syndrome cell adhesion molecule	
3	2	*Erbb4*	99,904,366	0.00203	0.00855	Pigmentation/Neurobehavioral functioning	[37]
4	3	*FCRL4*	13,339,357	0.00141	0.00317	Immunoglobulin	
5	3	*SLC35D1*	78,587,030	0.00061	0.00854	Responsible for skeletal dysplasia	[77]
6	3	*GRIK3*	109,293,546	0.00076	0.00761	Reward-related learning	[45]
7	4	*VWDE*	20,025,093	0.00918	0.07143	A carrier of clotting factor VIII (FVIII)	
8	4	*CAV1 & CAV2*	52,237,367	0.00092	0.02540	Cystic Fibrosis	
9	4	*TAS2R16*	88,279,974	0.00143	0.00887	Bitter taste receptor, type 2	
10	5	*CACNA1C*	109,068,036	0.48366	0.00013	Bipolar disorder and schizophrenia	[42]
11	6	*KIT/KDR*	72,087,087	0.00029	0.00011	Pigmentation	[30]
12	7	*OR*	15,246,693	0.00502	0.00194	Olfaction perception	
13	7	*OR*	43,810,382	0.00113	0.00905	Olfactory receptor family cluster 2	
14	7	*LOC783452*	52,837,137	0.66899	0.00589	Pro-neuregulin-2, membrane-bound isoform-like	
15	8	*OR13C8*	96,134,318	0.57280	0.00043	Olfactory receptor, family 13, subfamily C, member 8	
16	8	*ASTN2*	107,909,740	0.00058	0.00269	Astrotactin 2	
17	9	*LIN28B*	45,672,801	0.00982	0.00166	Cause gigantism and a delay in puberty	[78]
	9	*UNC93A*	103,514,079	0.0022	0.08933	Herpes simplex encephalitis type 1	
18	10	*ATL1*	43,656,593	0.00227	0.00557	Formation and growth of axons	
	11	*NRXN1*	33,156,535	0.00396	0.00018	Major depression	[43]
19	11	*LAMC3*	101,292,261	0.00222	0.00325	Cause malformations of occipital cortical development	[46]
20	11	*ADAMTS13*	104,415,591	0.00711	0.00736	Involved in blood clotting	
21	14	*MAGEA13PL*	42,271,706	0.00056	0.00178	Immune system	
22	14	*LOC100335199*	42,856,951	0.00034	0.00010	Tescalcin-like with unknown function	
23	15	*OR51A7*	50,749,853	0.87230	0.00048	Olfactory receptor, family 51, subfamily A, member 7	
24	16	*TNFRSF9*	46,579,025	0.00132	0.00207	Immune system	
25	17	*ULBP3*	40,044,391	0.00691	0.00917	“Sudden whitening of the hair” phenomenon	[40]
26	17	*TMEM132D*	48,769,405	0.00087	0.00021	Neurobehavioral functioning	[41]
27	18	*MC1R*	14,785,816	0.00785	0.00156	pigmentation	[35]
28	20	*STK10*	3,808,800	0.00119	0.00051		
29	21	*NRG4*	32,012,049	0.00368	0.00221	Pigmentation/Neurobehavioral functioning	[37]
30	21	*NPAS3*	44,609,230	0.34897	0.00137	High risk of developing schizophrenia	[44]
31	22	*MITF*	32,017,564	0.00172	0.00709	Pigmentation	[30]
32	25	*HS3ST4*	23,942,521	0.00494	0.03597	Involved in blood clotting	

1 Only best candidate genes are shown.

2 Position stands at the middle of the top hitch-hiked window in the candidate region.

Another candidate selective sweep was localized within a bitter taste receptor gene (*TAS2R16*, P_iHS_ = 0.00143, P_CLR_ = 0.00887) that enables animals to properly distinguish food sources and prevent them from ingesting potentially harmful compounds such as noxious defense compounds produced by plants [52]. It has been argued that elimination of the need to search for food in wild animals after their domestication as well as adaptation to new dietary habits may relax the evolutionary constraint acting on these genes [53]. Selection has possibly targeted *TAS2R16* as part of the new dietary habits emerging during cattle domestication.

#### Immune system and genetic disorders

Infectious diseases have been dominant threats to survival; therefore natural selection is expected to act strongly on innate immunity genes. Among the top selection candidates in our list are *MAGEA13P*-like (P_iHS_ = 0.00056, P_CLR_ = 0.00178) a member of melanoma-associated antigen family (see for instance Figure S7), *FCRL4* (P_iHS_ = 0.00141, P_CLR_ = 0.00317) an immunoglobulin receptor gene, *UNC93A* (P_iHS_ = 0.00220, P_CLR_ = 0.08933), associated with Herpes simplex encephalitis type 1, and finally *TNFRSF9* (P_iHS_ = 0.00132, P_CLR_ = 0.00207) induced by lymphocyte activation gene. Other noteworthy genes in our list are *CAV1* and *CAV2* (P_iHS_ = 0.00092, P_CLR_ = 0.02540) involved in Cystic Fibrosis, *DSCAM* (P_iHS_ = 0.00281, P_CLR_ = 0.00005) implicated in Down syndrome, *SLC35D1* (P_iHS_ = 0.00061, P_CLR_ = 0.00854) responsible for skeletal dysplasia and a strong signal on *Tescalin* like gene (P_iHS_ = 0.00034, P_CLR_ = 0.00010) with unknown function (Figure S8).

#### Blood coagulation

We found three genes annotated with blood clotting functions in the region of selection signals. The von Willebrand factor D and EGF domains (*VWDE*, P_iHS_ = 0.00918, P_CLR_ = 0.07143) a carrier of clotting factor VIII (FVIII), *ADAMTS13* (P_iHS_ = 0.00711, P_CLR_ = 0.00736) that cleaves *VWDE*, and heparan sulfate (glucosamine) 3-O-sulfotransferase 4 (*HS3ST4*, P_iHS_ = 0.00494, P_CLR_ = 0.03597) implicated in negative regulation of blood coagulation show signals of selection. This corresponds with some reports on primates along with human data suggesting potential signatures of positive selection for genes involved in blood clotting pathways [54], [55]. Further research on this group of genes would be required to address potential adaptation of blood coagulation genes in cattle.

There is a growing number of genome-wide scans for detecting historical positive selection in cattle and other farm animals. Previous studies in cattle however, have used low resolution panels of ascertained SNPs, mostly based on inter-population comparisons of site frequencies [18], [19], [20], [21], [22]. The notable candidate genes reported in these studies include *GHR*, *PDGFRA*, *KIT* and *MC1R* in association with body size and morphology traits in cattle. These signals generally differ from those reported by the Bovine HapMap consortium [7]. The most recent study based on 700k bovine array employed a composite framework that combines P-values from different tests across multiple breeds [56]. While the polymorphism content in both *KIT* and *MC1R* regions were under-represented for conducting an efficient selection scan, we found a poor overlap genome wide in comparison to our results. Besides different marker density and populations in both studies, the differences in the statistical approaches used could explain the discrepancy. The suggested statistical tests applied in this study recover selective events from different time periods and/or for different stages of the selective sweep (e.g., CLR vs. iHS). Furthermore, a selective sweep might be specific for one population and may not appear in other populations. Thus, combining results of multiple tests and across populations may mask real signals which could partly explain the low concordance with our single-breed single-test results.

### Validating putative sweeps with GWAS

**Figure 4:**
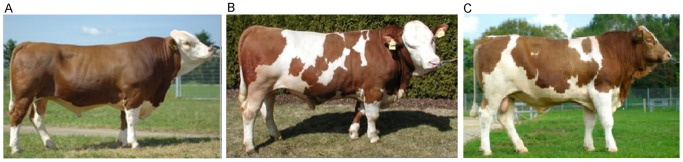
Fleckvieh animals with different coat coloring phenotypes. (A) without spot, (B) spotted and (C) red head. The figures were kindly supplied by BAYERN-GENETIK GmbH (http://www.fleckvieh.de).

If our candidate regions are in fact enriched for genes affected by selection related to domestication traits, they should overlap with regions identified in QTL mapping studies on these traits. This hypothesis was verified in an exemplary fashion for the complex trait coat color. Coat color in cattle is usually regarded to be a trait controlled by few loci of large effect [30]. There is a high degree of variation in color apparent within Fleckvieh population. Fleckvieh animals are phenotypically characterized by being red, spotted or not, and having white legs and a white head, animals with red head occasionally occur but are considered as a deviation from the breed standard. We used the coat color traits recorded respectively as the proportion of daughters of bulls ‘without spotting’ and with ‘red head’ to validate putative sweeps for coloration phenotypes (Figure 4).

**Figure 5:**
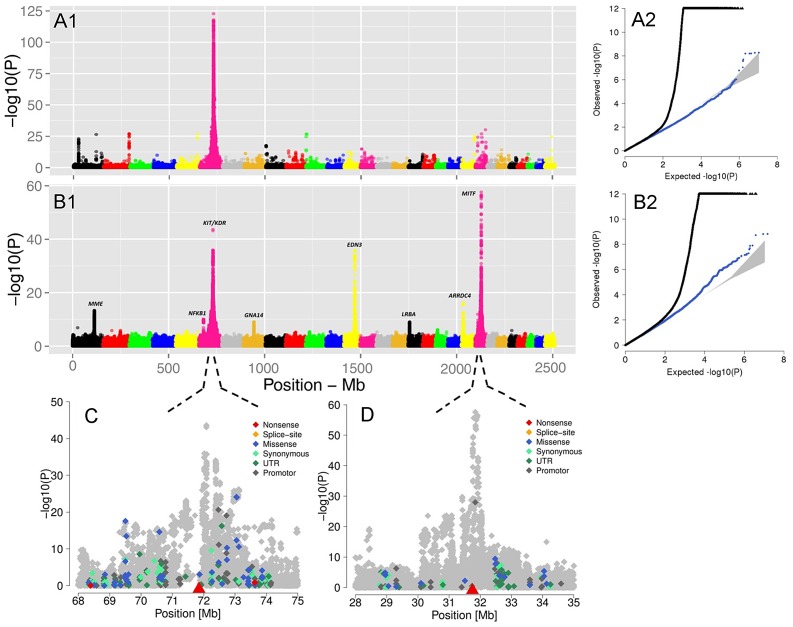
The visualization of the signals revealed by association analyses for coat coloring traits. GWAS are presented for the proportion of daughters with red head (A1) and the proportion of daughters without spotting (B1) based on 15,182,131 imputed variants in 3062 Fleckvieh animals. In (B1) the largest effects emerge from eight SNPs summarized in Table 2 with MITF (P = *2.65e-58*) and *KIT/KDR* (P = 2.46e-44) at the top. Together, *KIT/KDR* and MITF explained 36.25% of the residual variance of the trait in the studied population. A2 and B2 are the corresponding quantile-quantile plots. Shown in blue is the quantile-quantile plot resulting from removal of all SNPs in the region of significant genes listed in Table 2, for both traits. The shaded area is the 95% concentration band under the null hypothesis of no association. Panels C and D are detailed overviews of the associated regions on BTA6 and BTA22, respectively. Variants in the promoter (defined to encompass 1,000 bp upstream of the transcription start), in the untranslated regions (UTR) and in the amino-acid coding region are highlighted with different color. The red triangles indicate the genomic positions of KIT and MITF genes.

We performed a Genome Wide Association Study (GWAS) on 3602 animals for which genotypes of 15,182,131 SNPs were imputed (see Material and Methods). The GWAS revealed eight SNPs with large effect on the ‘proportion of daughters without spotting’ (Figure 5B1). The SNP with the most significant effect is a non-genic variant in the vicinity of the *MITF* (P = 2.65e-58) gene on BTA22. Another highly significant but non-genic SNP coincides with a selection candidate region (e.g., Figure 3C) covering the *KIT* and *KDR* (P = 2.46e-44) loci. There are also two significant signals, respectively next to endothelin 3 (*EDN3*) (P = *2.42e-36*) and an uncharacterized protein at the proximity of the membrane metallo-endopeptidase (*MME*) gene (P = *5.11e-14*). *EDN3* plays a significant role during the early development of melanocytes in their response to ultraviolet radiation, and in pathological conditions including melanoma. Severe pigmentation defects of mutations in *EDN3* in mice, human and chicken are well-described (for review see [57]). In human models, *MME* is expressed at the surface of melanoma cells and are involved in the regulation of melanogenesis [58]. Although these SNPs were the top association signals for each of the identified QTL, distinguishing causal variants from nearby neutral loci may be the most difficult issue, as those variants possibly stay in LD with the actual selected locus that may produce similar signals due to genetic hitch-hiking.

Genomic relationship matrices were built separately for each chromosome and QTL using imputed variants. The phenotypic variation explained by each chromosome/QTL was then estimated with the effects of all chromosomes/QTL fitted simultaneously using GCTA [59]. All together, the imputed variants explained 82.37% of the phenotypic variation (i.e. 94% of the heritability) (Figure S9). Together, the eight identified QTL explained 49.76% of the phenotypic variation (i.e. 56.8% of the heritability).

The GWAS for the ‘proportion of daughters with red head’ was modeled in the same way. Visualizing of results shows a different genetic control for the red head phenotype when is compared to the spotting. The strongest GWAS revealed signal (*P* = 6.8e-125) was on the *KIT* locus on BTA6 (Figure 5A1), which alone explained 34.81% of the total variation of the trait (Table 2).

**Table 2:** A descriptive summary of GWA studies for coat color variation in Fleckvieh animals.

Chr	Position (bp)	Top-SNP(NCBI reference ID)	Minor allele frequency	P-Value	Candidate gene	Proportion of EBV variation explained [%]
**Without spotting**						
1	113,261,262	rs110220767	0.27	5.11e-14	*MME*	2.95
6	23,655,204	rs43461001	0.16	8.89e-11	*NFKB1*	1.26
6	72,085,585	rs135123206	0.07	2.46e-44	*KIT/KDR*	14.68
8	53,907,785	rs110041961	0.35	1.01e-9	*GNA14*	0.8
13	57,580,515	rs382817429	0.17	2.42e-36	*EDN3*	5.75
17	7,202,981	rs135947957	0.42	1.04e-9	*LRBA*	0.92
21	9,225,382	rs42261960	0.37	6.65e-17	*ARRDC4*	1.83
22	31,841,994	rs41642495	0.12	2.65e-58	*MITF*	21.57
**Red Head**						
6	71,404,818	rs137525659	0.10	6.8e-108	*KIT*	34.81

Previous GWAS on coat color in cattle have identified polymorphism in *MC1R*, *KIT* and *MITF* [35], . In both GWA analyses in this study, the *KIT* locus is associated, but *MITF*, *EDN3* and *MME* are related only with spotting phenotype. This observation suggests that a different genetic mechanism regulates these phenotypes in the Fleckvieh breed. Recently, Pausch et al. [60] studied the genetics controlling the peculiar pigmentation surrounding the eyes in Fleckvieh animals using the 700K SNP panel. They found a strong association between *MITF* and *KIT* genes with the UV-protective eye area pigmentation in Fleckvieh cattle, but no association with *EDN3* and *MME* genes. Comparing GWAS results from coat spotting, red head, and eye area pigmentation reveals a complex genetic background for the different pigmentation phenotypes in the Fleckvieh population.

To test for an overlap between the selection candidates and the QTL study, we performed two randomization experiments on coat spotting phenotype using either single SNPs or window-based estimates of |iHS| versus P-values from GWAS (see Methods). Both randomizations revealed no single randomized dataset more extreme than the real data.

While there is no association detected for *MC1R* gene in the GWAS, selection signature analyses suggests that there has been a selective sweep at or near the *MC1R* gene on BTA18 (Figure S2A). *MC1R* variants have been shown to alter pigment synthesis in a range of species, as well as coat color spotting in pigs [61]. At least three major alleles exist in cattle *MC1R*, the *E^+^* wild type, *E^D^* dominant black locus, and *e* recessive red locus [35]. Since the red variant is fixed in Fleckvieh population, no [possible] association with coat spotting or red head can be traced through GWAS. This also explains why the locus is not very extreme when explored by the iHS statistic (see Figure S2A). We postulate that red hair has been under very recent selection (e.g., human driven selection during breed formation) resulting in fixation of the red variant in the Fleckvieh cattle, while simultaneously fixing alleles at nearby hitch-hiked loci. To validate this hypothesis, we calculated SNP-specific F_ST_ [62] for 1,173 SNPs on BTA18 which have been genotyped in 2,084 Holstein-Friesian (black and white coat color) and 2,539 Fleckvieh (red and white coat color) animals using 50K SNP arrays. This revealed a strong differentiation hit located at 14.42 Mb in immediate vicinity to *MC1R*, further supporting findings of selection analyses (Figure S2B). Therefore, our results exemplify how a historical selective sweep that underwent fixation can be localized by employing a population genetic approach.

**Figure 6:**
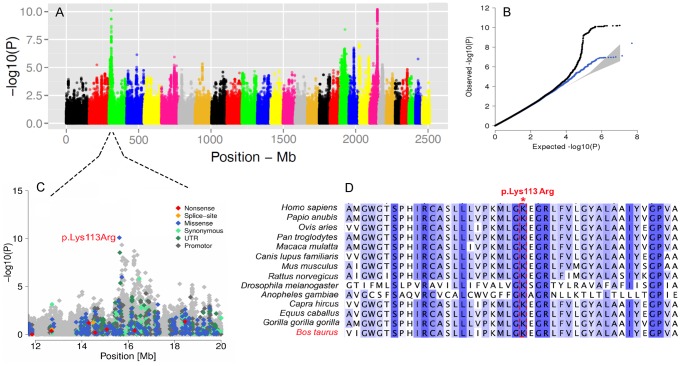
The visualization of the signals revealed by association analyses for SCC trait. Manhattan plot presents the association of 15'182'131 imputed SNPs with the “somatic cell count” trait in 3602 Fleckvieh animals (A). Panel B represents corresponding quantile-quantile plot. Shown in blue is the quantile-quantile plot resulting from excluding SNPs in the region of significant genes. The shaded area represents 95% concentration band under the null hypothesis of no association. Panel C displays the associated region on BTA3 with a higher resolution. Variants in the promoter (defined to encompass 1,000 bp upstream of the transcription start), in the untranslated regions (UTR) and in the amino-acid coding region are highlighted with different color. Panel D provides a multi species alignment of *DC-STAMP* domain containing 1 encoded by *DCST1* gene.

We further conducted GWA studies for somatic cell count (SCC, Figure 6A) and body size (Figure S10) based on daughter-derived phenotypes (estimated breeding values, EBVs) for these traits. In cattle breeding schemes, somatic cell count measured as the log number of somatic cells per *ml* of milk is widely used as an indicator for incidence of mastitis. The GWAS for SCC revealed strong association on BTA22 nearby the *LTF* gene encoding lactotransferrin (Table S5). Due to its antimicrobial and anti-inflammatory activity [63], *LTF* is a strong candidate for mastitis resistance. The most significant SNP is located ∼8 Kb upstream of the translation start of *LTF* and might affect expression of *LTF* during mammary gland infections [64]. Another QTL is located on BTA3 and the most significant SNP (rs386094483, P = 7.95e-11) is a Lysine-to-Arginine substitution in the *DC-STAMP* domain containing 1-encoding gene (*DCST1*, p.K113R, Chr3:15613949) (Figure 6C). *DCST1* plays an important role in the initiation of the immune system by antigen processing [65], [66], [67]. The affected amino acid is highly conserved among species and is a strong candidate causal mutation for the somatic cell count in cattle (Figure 6D).

The GWAS for body size revealed three QTLs. One example is the *PLAG1* region on BTA14 which is significantly associated with body size (P = 1.5e-27). This region was already shown to affect growth-related traits in several species including cattle [68]. This QTL is also associated with the calving difficulties and an increased stillbirth rate in cattle, probably resulting from an enhanced fetal growth [69].

Table S5 presents a descriptive summary of the most significant SNPs and the proportion of EBV variance attributable to the five identified QTL regions. Altogether, the imputed variants explain 81.35% and 80.73% of the variation of EBV, respectively for the SCC and body size traits. A schematic illustration of chromosomal contributions for the total variance of these traits are represented respectively, in Figures S11 and S12.

In contrast to regions associated with color variation QTLs, these regions show no overlap with selection candidates. One possible explanation is that most selection affecting body size and somatic cell count has affected alleles that are no longer segregating in this breed. This raises the question as to why the currently segregating alleles affecting these traits do not show strong signals of selection. Possibly, the alleles that are still segregating, even after intense artificial selection during domestication, may have negative pleiotropic effects preventing them from increasing in frequency in the population. Additionally, selection is likely to have affected standing variation. If the selected mutations were segregating on multiple different haplotypes before selection started, both the iHs and the CLR statistic may have limited power to detect selection.

We present the first comprehensive study for localizing signatures of past selection in cattle based on full re-sequencing data. 106 candidate regions were identified containing genes with biological functions involved in blood clotting, immune-defense functions, pigmentation pattern, sensory perceptions and neurobehavioral functioning. The detection of genes related to pigmentation is not surprising since a specific coat color pattern, such as red (in various shadings) coat with white head, is constitutive for the breed definition of Fleckvieh and therefore fundamental for the breed formation process. Using the same samples, we also performed a coat color GWA study, and show that there is a strong overlap between genes identified in the GWAS and in the selection scan. As demonstrated for the gene MC1R, selection signatures can be detected in regions where anthropogenic selection has fixed the desired allele and, consequently, GWAS fails. This illustrates the potential for population genetic techniques to identify genomic regions relating to phenotypes of importance to breeders. Comparing GWAS results for different traits also provides further evidence regarding the complexity of genetics underlying coat coloring in cattle.

## Materials and Methods

### Ethics statement

DNA needed for the study was previously extracted from commercial AI bull semen straws. No ethics statement is thus required.

### Sequenced-based imputation

For the purpose of this study we used data from Jansen et al. [23]. Briefly, it consists low to medium coverage (∼7.4-fold) sequence of the entire genomes of 43 key and contemporary animals representing ∼69% of the genetic diversity of the current German Fleckvieh population.

The sequence panel consisting of 15,182,131 SNPs with an average inter-marker space equal to 178±115 bp was used for a two-step imputation using default setting in Beagle [70] and Minimac [71], respectively. Imputation started from a medium density panel (50K SNPs) bridged by a high density panel (700K SNPs) to the full sequences using 43 reference animals. We evaluated the accuracy of sequence-based imputation for chromosomes 5, 15 and 25 within the high density panel (700K SNPs). Genotypes for randomly selected 66% of the SNPs were retained, while genotypes for the remaining SNPs were masked to mimic missing genotypes. Those were imputed using Beagle and Minimac (see above) based on sequence-derived genotypes of 43 re-sequenced animals. Imputation accuracy was assessed as the correlation between array-derived and imputed genotypes. This approach yielded high imputation accuracy for frequent alleles (e.g., MAF >5%) (Figure S13). However, the number of re-sequenced animals (n = 43) might not be sufficient for imputing low-frequency variants with a sophisticated accuracy, which agrees with a previous report in cattle [72].

The individual call-rate was >95% for all animals genotyped with SNP arrays. After quality control (call-rate per SNP >95%, minor allele frequency >0.5%, no significant deviation of the Hardy-Weinberg-Equilibrium (P>10-6), known chromosomal position), the medium and high density dataset comprised genotypes for 39,304 (n = 2,309) and 645,189 SNPs (n = 1,293), respectively.

### Linkage disequilibrium

We quantified LD using the squared correlation coefficients (*r^2^*) between pairs of SNPs. Evaluations of SNP to SNP pairwise *r^2^* were completed based on the panels of 15,000 SNPs randomly sampled across two segments of each 5 Mb on the chromosomes 5, 10, 15, 20 and 26. We then compared measures of *r^2^* to those of 50K and 700K bovine arrays across the corresponding chromosomes.

### Detecting positive selection

Evidence of positive selection was investigated through multiple statistics: We performed the Composite Likelihood Ratio test (CLR) using information from allele frequencies to detect a completed sweep [11]. Briefly, CLR relies on identifying skews in the allele frequency spectrum toward excess of rare and frequent alleles. To infer ongoing sweeps we employed the integrated Haplotype Homozygosity Score (iHS) that explores the structure of haplotype and essentially indicates unusually long haplotypes carrying the ancestral and derived allele [12]. Single site values for iHS were averaged in non-overlapping windows of 40 Kb across the genome resulting in a total of 62'196 windows. Window size was adapted based on the extent of LD as discussed above (Figure 2). The variance of SNP statistics within 40 kb windows (i.e., Var|iHS| = 0.27) was significantly smaller than that among randomly selected SNPs (i.e., Var|iHS| = 0.35), confirming that the windows effectively grouped SNPs with more similar statistic values. Figure S14 visualizes the number of SNPs distributed across sliding windows. To produce comparable results of the composite likelihood ratio (CLR) test, which is a multi-locus statistic, the grid size was taken as 40 kb. This resulted in 62,788 local CLR values across the genome. The empirical P-values were generated by genome wide ranking of |iHS| and CLR values. The list of |iHS| and CLR scores for all windows are available in supporting material as Dataset S1 and Dataset S2, respectively. The iHS metric was calculated using the R package ‘rehh’ [73]. For estimating CLRs we used ‘sweepfinder’ [11] with a background allele frequency spectrum calibrated genome-wide. We further used custom programs to calculate the ‘observed heterozygosity’ (Het), which should be reduced in regions affected by a sweep [74], Tajima's D [8], Fay and Wu H statistic [9], and the number of Segregating Loci (nSL), a recently developed statistic related to iHS in Rasmus Nielsen's Lab. The metrics were estimated using different window size to explore the sensitivity to the choice of window. We annotated candidate genomic regions by aligning the positions to the bovine genome sequence assembly build 6.1, to reveal genes and ESTs located in the respective region.

### Randomization test

To test if the overlap between a selection scan and a GWAS is significantly different from that expected at random, we performed permutation tests. We generated 10,000 simulated data sets by permuting p-values among either single SNPs or windows. For example, we calculated empirical p-values for windows based on |iHS|, then counted the number of instances where a GWAS association (p-value<10^−6^, Bonferroni corrected threshold) overlaps a high-scoring (top 1%) selection window. The observed value of the overlap statistic was then compared to the distribution of overlap statistics in the permuted data sets.

### Sequenced-based association study

After imputation, genotypes of 15,182,131 SNPs in 3602 individuals were used for a GWAS for four different traits: the square-root transformed proportion of daughters without spotting and with red head and estimated breeding values for somatic cell counts and body size. EMMAX [75] was used to fit the model 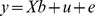 , where 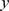 is the vector of phenotypes, 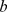 is the SNP effect, 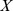 is a design matrix of allele dosages for the imputed SNPs, 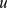 is the additive genetic effect 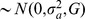 , where 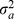 is the additive genetic variance, 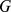 is the realized genomic relationship matrix estimated using genotype information [76], and 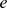 is the random residual term.

## Supporting Information

Figure S1 Evidence for the effects of MAF (A) and sample composition (B) on the strength of LD. Panel A compares LD from sequence (n = 43, red) and array (n = 1,293, green) data against SNP set with MAF >0.2 (n = 43, blue). In Panel B, LD curves from sequence (red) and array (green) data are compared against set of bovine 700K SNPs sub-selected from only sequences of only 43 individuals.
(TIF)

Figure S2 Selection for coat coloring. Panel A is detailed schematic illustration of the region harboring *MC1R* locus in Fleckvieh animals. The pattern of composite likelihood ratio (CLR), |iHS|, standardized heterozygosity (ZHet), Tajima D, number of Segregating Loci and Fay and Wu H values are depicted. The multi-locus CLRs are estimated in grid size = 5 Kb while other metrics are single SNP values accumulated in windows of 40 Kb and depicted in steps of 5 Kb. *MC1R* is located between 14,757,332 and 14,759,082 bp on BTA18. Panel B displays sliding 3-SNP windows of F_ST_ calculated between Holstein-Friesian (black coat color) and Fleckvieh (red coat color) animals based on 1173 SNPs on BTA18. The dashed line displays top 0.1% cutoff and the red triangle represents the position of *MC1R*.
(TIF)

Figure S3 A detailed schematic illustration of the region harboring *NRG4* locus in Fleckvieh animals. The pattern of composite likelihood ratio (CLR), |iHS|, standardized heterozygosity (ZHet), Tajima D, number of Segregating Loci and Fay and Wu H values are depicted. The multi-locus CLRs are estimated in grid size = 5 Kb while other metrics are single SNP values accumulated in windows of 40 Kb and depicted in steps of 5 Kb. *NRG4* is located between 31,824,897 and 31,825,956 bp on BTA21.
(TIF)

Figure S4 A detailed schematic illustration of the region harboring *Erbb4* locus in Fleckvieh animals. The pattern of composite likelihood ratio (CLR), |iHS|, standardized heterozygosity (ZHet), Tajima D, number of Segregating Loci and Fay and Wu H values are depicted. The multi-locus CLRs are estimated in grid size = 5 Kb while other metrics are single SNP values accumulated in windows of 40 Kb and depicted in steps of 5 Kb. *Erbb4* is located between 99,660,620 and 100,632,794 bp on BTA2.
(TIF)

Figure S5 A detailed schematic illustration of the region harboring *TMEM132D* locus in Fleckvieh animals. The pattern of composite likelihood ratio (CLR), |iHS|, standardized heterozygosity (ZHet), Tajima D, number of Segregating Loci and Fay and Wu H values are depicted for the region harboring *TMEM132D* locus in Fleckvieh animals. The multi-locus CLRs are estimated in grid size = 5 Kb while other metrics are single SNP values accumulated in windows of 40 Kb and depicted in steps of 5 Kb. *TMEM132D* is located between 48,199,004 and 49,090,245 bp on BTA17.
(TIF)

Figure S6 A detailed schematic illustration of the region harboring *GRIK3* locus in Fleckvieh animals. The pattern of composite likelihood ratio (CLR), |iHS|, standardized heterozygosity (ZHet), Tajima D, number of Segregating Loci and Fay and Wu H values are depicted. The multi-locus CLRs are estimated in grid size = 5 Kb while other metrics are single SNP values accumulated in windows of 40 Kb and depicted in steps of 5 Kb. *GRIK3* is located between 109,422,556 and 109,667,304 bp on BTA3.
(TIF)

Figure S7 A detailed schematic illustration of the region harboring *OLIG1* locus in Fleckvieh animals. The pattern of composite likelihood ratio (CLR), |iHS|, standardized heterozygosity (ZHet), Tajima D, number of Segregating Loci and Fay and Wu H values are depicted. The multi-locus CLRs are estimated in grid size = 5 Kb while other metrics are single SNP values accumulated in windows of 40 Kb and depicted in steps of 5 Kb. *OLIG1* is located between 1,775,224 and 1,777,299 bp on BTA1.
(TIF)

Figure S8 A detailed schematic illustration of the regions harboring two candidate selective sweeps harboring *MAGEA13P*-like and tescalcin-like loci. The pattern of composite likelihood ratio (CLR), |iHS|, standardized heterozygosity (ZHet), Tajima D, number of Segregating Loci and Fay and Wu H values are depicted. *MAGEA13P*-like and tescalcin-like loci are located, respectively on 42,269,085..42,270,099 bp and 42,686,629..42,687,350 bp on BTA14. The multi-locus CLRs are estimated in grid size = 5 Kb while other metrics are single SNP values accumulated in windows of 40 Kb and depicted in steps of 5 Kb.
(TIF)

Figure S9 Chromosomal partitioning of the phenotypic variance of the ‘proportion of daughters without spotting’. The grey and blue bars indicate the fraction of phenotypic variance attributed to a particular chromosome and QTL region, respectively. The triangles represent the cumulative proportion of phenotypic variance attributable to the 30 chromosomes.
(TIF)

Figure S10 Manhattan plot of association of 15'182'131 imputed variants with the body size in 3602 Fleckvieh animals (A). Panel B represents corresponding quantile-quantile plot. Shown in blue is the quantile-quantile plot resulting from excluding SNPs in the region of significant genes. The shaded area represents 95% concentration band under the null hypothesis of no association.
(TIF)

Figure S11 Chromosomal partitioning of the EBV variance of the ‘somatic cell count’ trait. The grey and blue bars indicate the fraction of phenotypic variance attributed to a particular chromosome and QTL region, respectively. The triangles represent the cumulative proportion of phenotypic variance attributable to the 30 chromosomes.
(TIF)

Figure S12 Chromosomal partitioning of the EBV variance of the ‘body size’ trait. The grey and blue bars indicate the fraction of phenotypic variance attributed to a particular chromosome and QTL region, respectively. The triangles represent the cumulative proportion of phenotypic variance attributable to the 30 chromosomes.
(TIF)

Figure S13 Evaluation of imputation accuracy. Correlation between imputed and array-derived genotypes as a function of the minor allele frequency. Genotypes for randomly selected SNPs were set to missing and subsequently imputed based on sequence-derived genotypes of 43 re-sequenced animals.
(TIF)

Figure S14 Distribution of the number of SNPs in 62'196 non-overlapping windows along BTA1 to BTA29.
(TIF)

Table S1 Comparison of fraction (%) of marker pairs with different r2 levels as a function of distance (kb).
(DOCX)

Table S2 Summary statistics of testing the difference between sequence vs., array based LD.
(DOCX)

Table S3 Candidate regions identified by iHS analysis.
(DOCX)

Table S4 Candidate regions identified by CLR analysis.
(DOCX)

Table S5 Results of GWA studies based on estimated breeding values for Somatic cell count and body size in Fleckvieh animals.
(DOCX)

Dataset S1 Genome wide iHS scores.
(CSV)

Dataset S2 Genome wide CLR scores.
(CSV)
