# Supplementary figures and images for: Classic Selective Sweeps Revealed by Massive Sequencing in Cattle
Source: PLoS Genet. 2014 Feb 27;10(2):e1004148. doi: 10.1371/journal.pgen.1004148 (PMC3937232; doi:10.1371/journal.pgen.1004148)

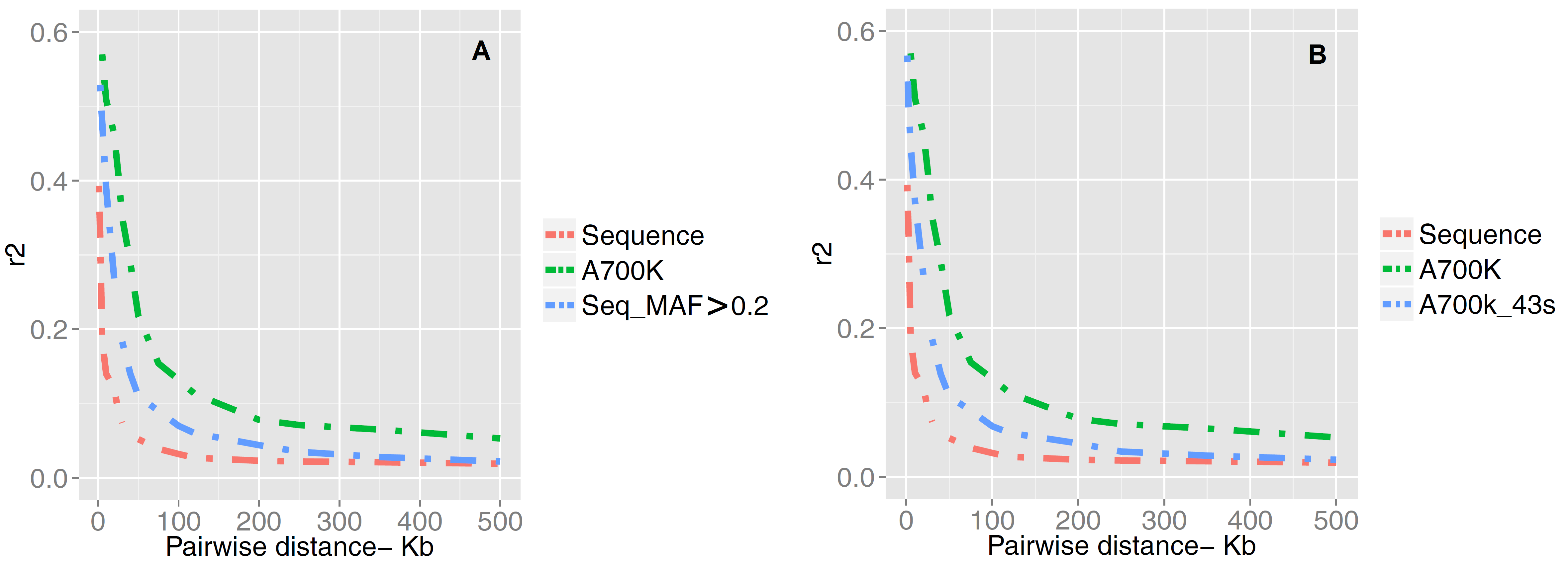

Supplement: Figure S1 — Evidence for the effects of MAF (A) and sample composition (B) on the strength of LD. Panel A compares LD from sequence (n = 43, red) and array (n = 1,293, green) data against SNP set with MAF >0.2 (n = 43, blue). In Panel B, LD curves from sequence (red) and array (green) data are compared against set of bovine 700K SNPs sub-selected from only sequences of only 43 individuals. (TIF) [file pgen.1004148.s001.tif]

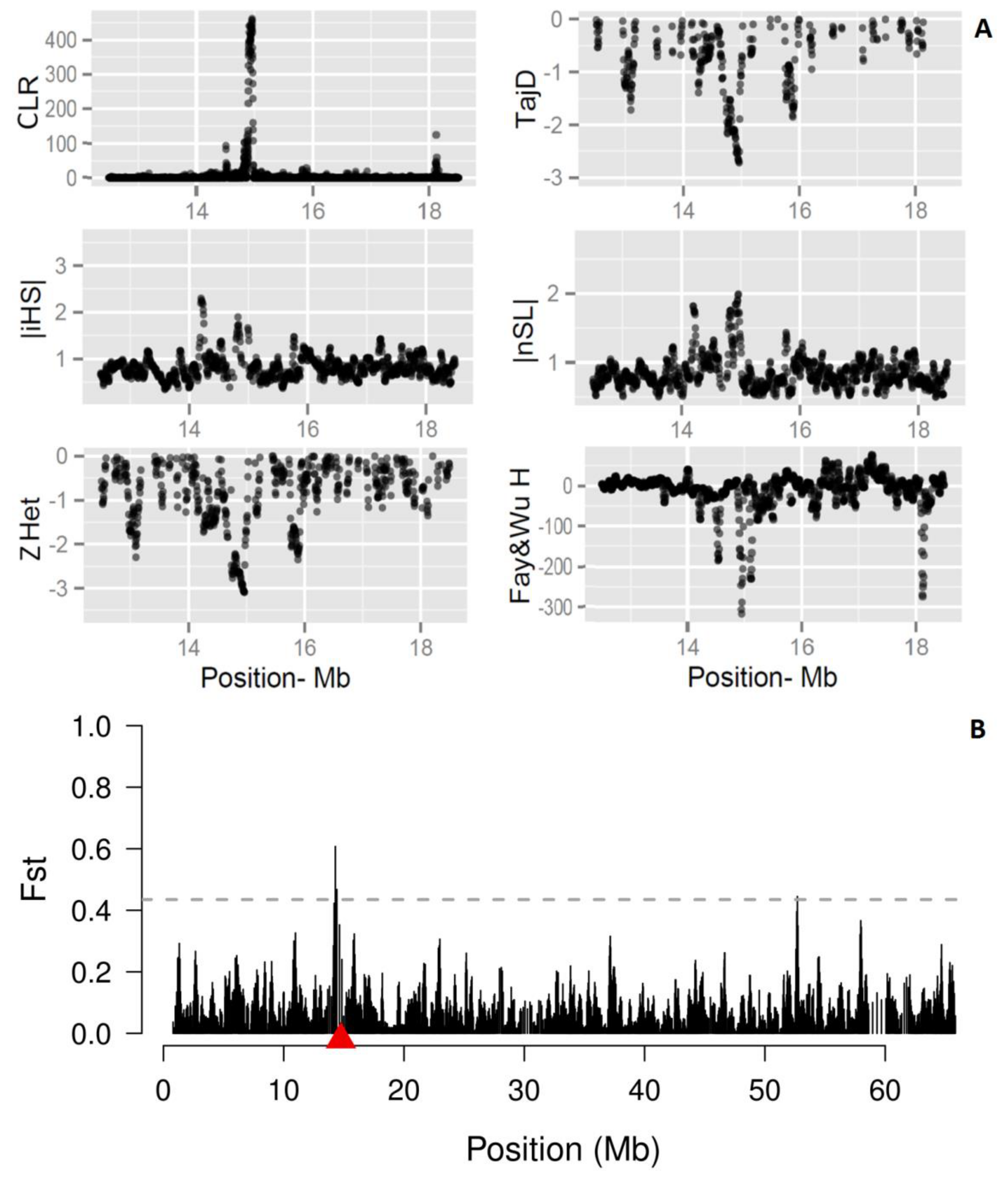

Supplement: Figure S2 — Selection for coat coloring. Panel A is detailed schematic illustration of the region harboring MC1R locus in Fleckvieh animals. The pattern of composite likelihood ratio (CLR), |iHS|, standardized heterozygosity (ZHet), Tajima D, number of Segregating Loci and Fay and Wu H values are depicted. The multi-locus CLRs are estimated in grid size = 5 Kb while other metrics are single SNP values accumulated in windows of 40 Kb and depicted in steps of 5 Kb. MC1R is located between 14,757,332 and 14,759,082 bp on BTA18. Panel B displays sliding 3-SNP windows of FST calculated between Holstein-Friesian (black coat color) and Fleckvieh (red coat color) animals based on 1173 SNPs on BTA18. The dashed line displays top 0.1% cutoff and the red triangle represents the position of MC1R. (TIF) [file pgen.1004148.s002.tif]

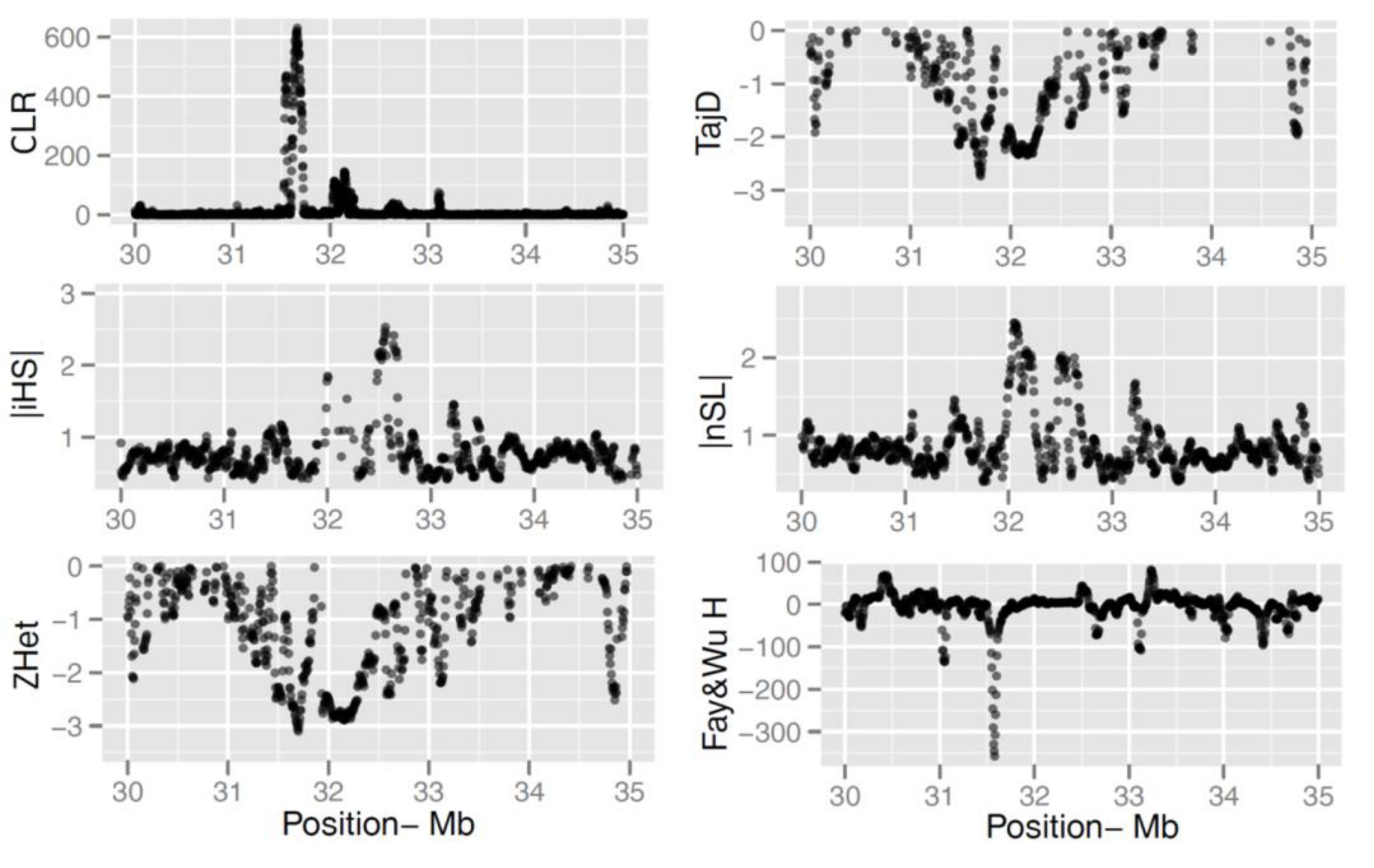

Supplement: Figure S3 — A detailed schematic illustration of the region harboring NRG4 locus in Fleckvieh animals. The pattern of composite likelihood ratio (CLR), |iHS|, standardized heterozygosity (ZHet), Tajima D, number of Segregating Loci and Fay and Wu H values are depicted. The multi-locus CLRs are estimated in grid size = 5 Kb while other metrics are single SNP values accumulated in windows of 40 Kb and depicted in steps of 5 Kb. NRG4 is located between 31,824,897 and 31,825,956 bp on BTA21. (TIF) [file pgen.1004148.s003.tif]

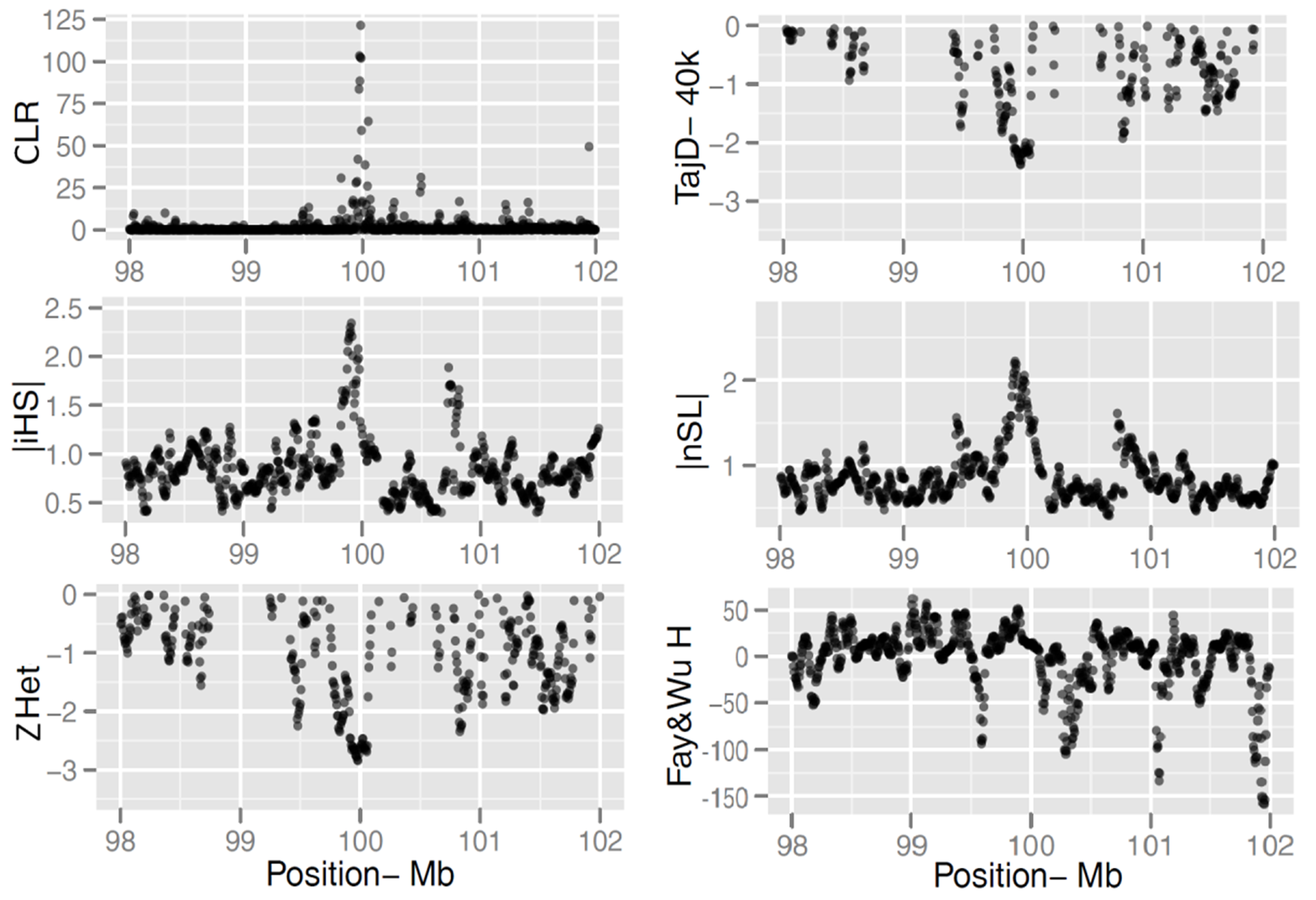

Supplement: Figure S4 — A detailed schematic illustration of the region harboring Erbb4 locus in Fleckvieh animals. The pattern of composite likelihood ratio (CLR), |iHS|, standardized heterozygosity (ZHet), Tajima D, number of Segregating Loci and Fay and Wu H values are depicted. The multi-locus CLRs are estimated in grid size = 5 Kb while other metrics are single SNP values accumulated in windows of 40 Kb and depicted in steps of 5 Kb. Erbb4 is located between 99,660,620 and 100,632,794 bp on BTA2. (TIF) [file pgen.1004148.s004.tif]

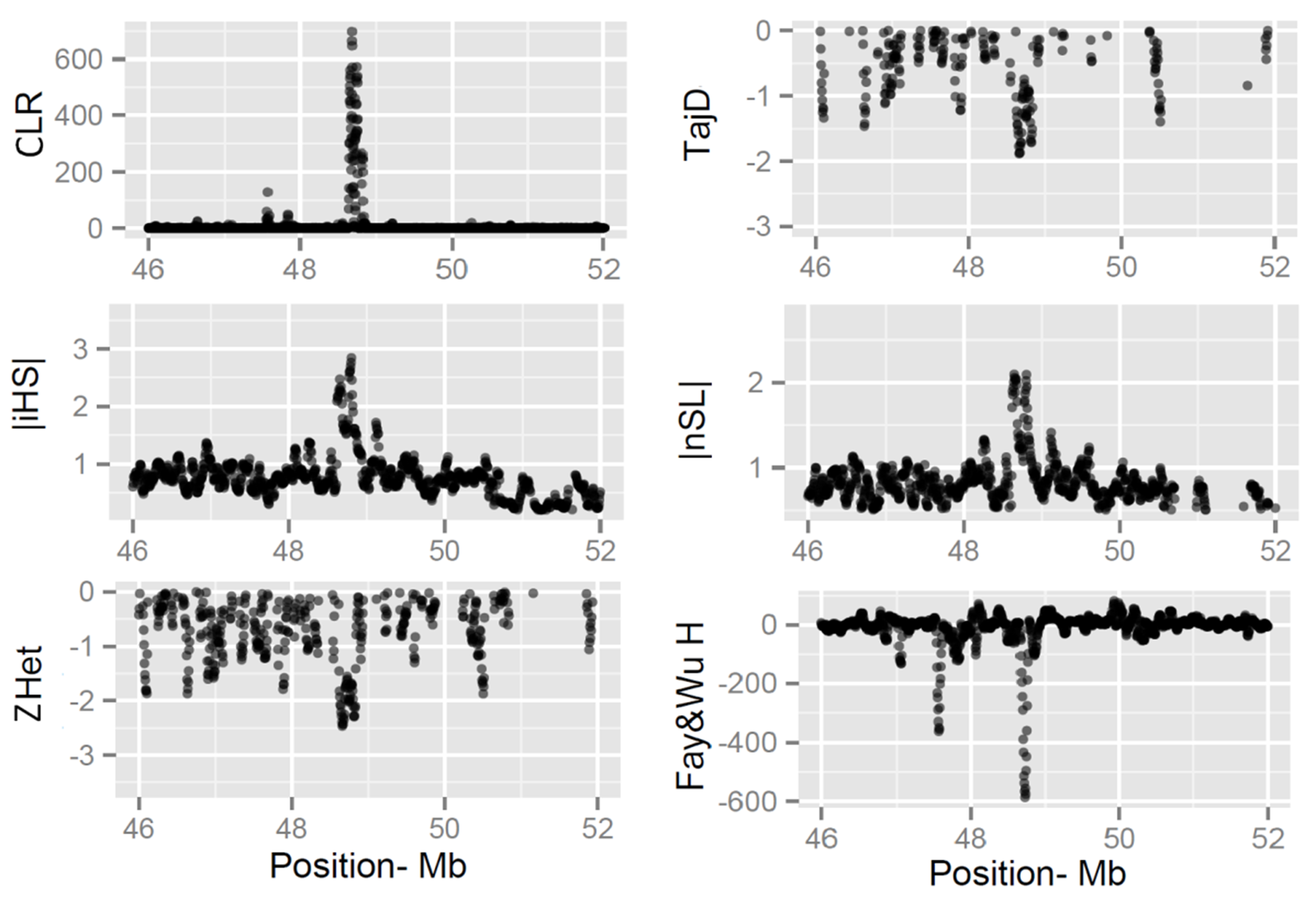

Supplement: Figure S5 — A detailed schematic illustration of the region harboring TMEM132D locus in Fleckvieh animals. The pattern of composite likelihood ratio (CLR), |iHS|, standardized heterozygosity (ZHet), Tajima D, number of Segregating Loci and Fay and Wu H values are depicted for the region harboring TMEM132D locus in Fleckvieh animals. The multi-locus CLRs are estimated in grid size = 5 Kb while other metrics are single SNP values accumulated in windows of 40 Kb and depicted in steps of 5 Kb. TMEM132D is located between 48,199,004 and 49,090,245 bp on BTA17. (TIF) [file pgen.1004148.s005.tif]

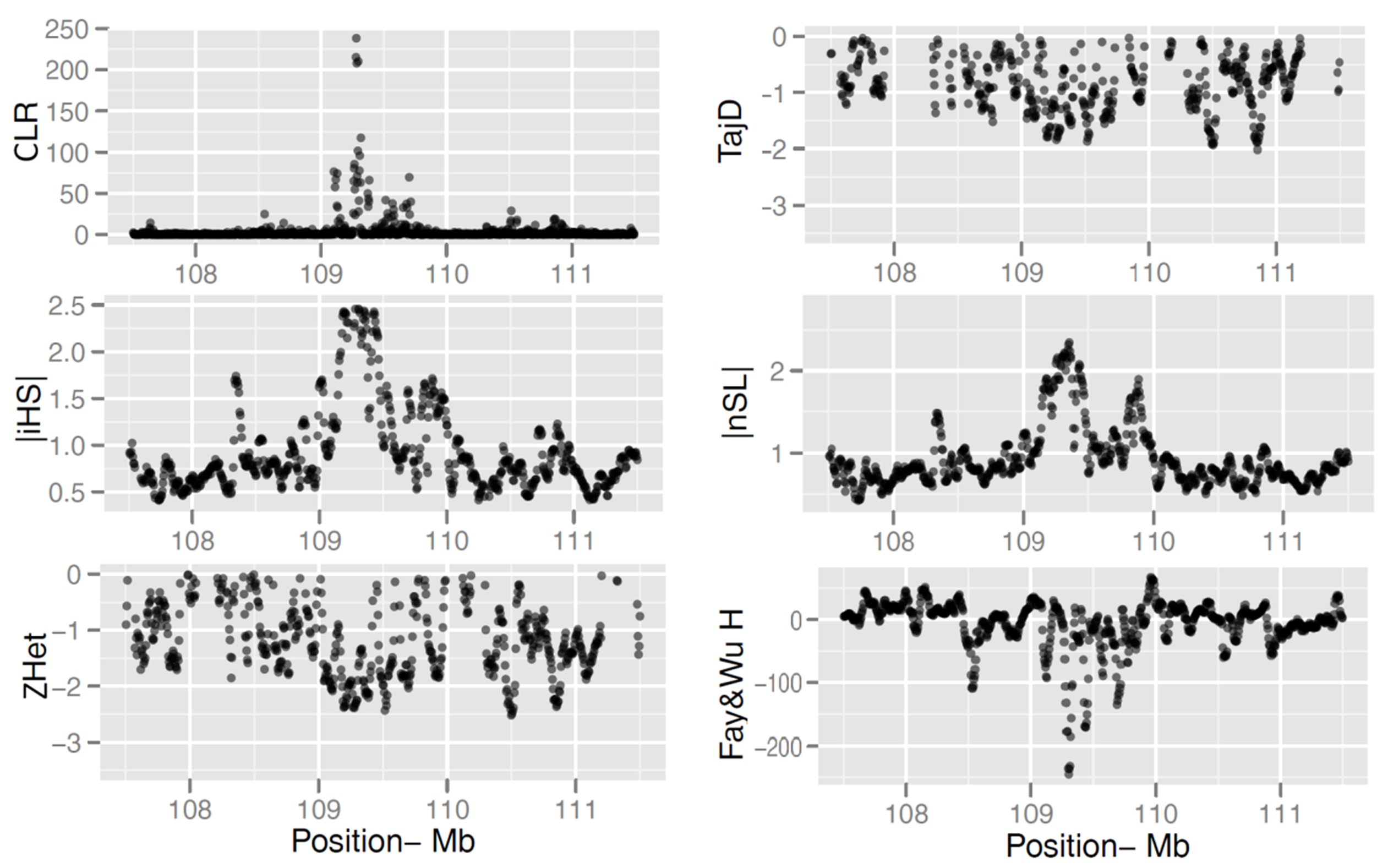

Supplement: Figure S6 — A detailed schematic illustration of the region harboring GRIK3 locus in Fleckvieh animals. The pattern of composite likelihood ratio (CLR), |iHS|, standardized heterozygosity (ZHet), Tajima D, number of Segregating Loci and Fay and Wu H values are depicted. The multi-locus CLRs are estimated in grid size = 5 Kb while other metrics are single SNP values accumulated in windows of 40 Kb and depicted in steps of 5 Kb. GRIK3 is located between 109,422,556 and 109,667,304 bp on BTA3. (TIF) [file pgen.1004148.s006.tif]

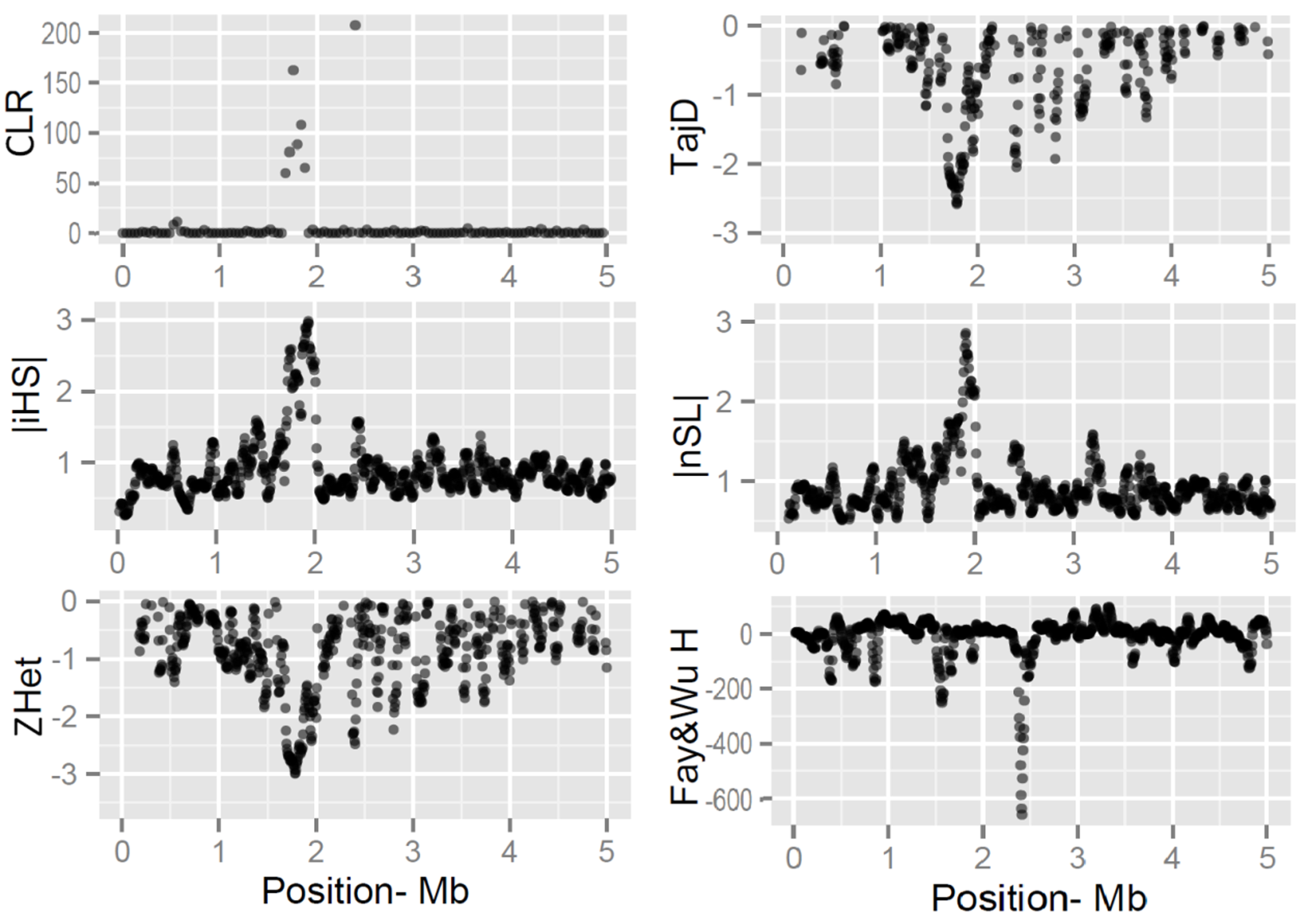

Supplement: Figure S7 — A detailed schematic illustration of the region harboring OLIG1 locus in Fleckvieh animals. The pattern of composite likelihood ratio (CLR), |iHS|, standardized heterozygosity (ZHet), Tajima D, number of Segregating Loci and Fay and Wu H values are depicted. The multi-locus CLRs are estimated in grid size = 5 Kb while other metrics are single SNP values accumulated in windows of 40 Kb and depicted in steps of 5 Kb. OLIG1 is located between 1,775,224 and 1,777,299 bp on BTA1. (TIF) [file pgen.1004148.s007.tif]

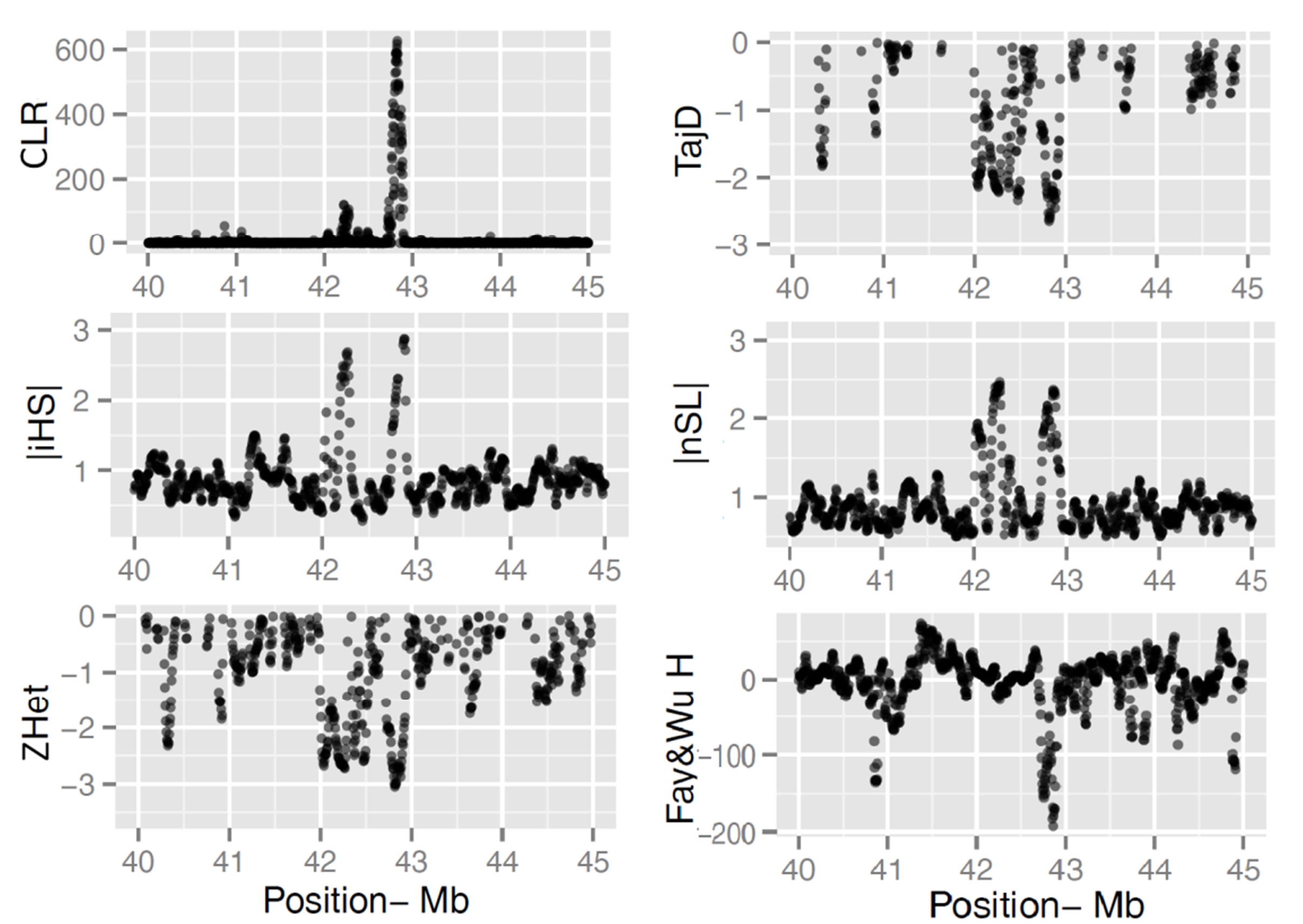

Supplement: Figure S8 — A detailed schematic illustration of the regions harboring two candidate selective sweeps harboring MAGEA13P-like and tescalcin-like loci. The pattern of composite likelihood ratio (CLR), |iHS|, standardized heterozygosity (ZHet), Tajima D, number of Segregating Loci and Fay and Wu H values are depicted. MAGEA13P-like and tescalcin-like loci are located, respectively on 42,269,085..42,270,099 bp and 42,686,629..42,687,350 bp on BTA14. The multi-locus CLRs are estimated in grid size = 5 Kb while other metrics are single SNP values accumulated in windows of 40 Kb and depicted in steps of 5 Kb. (TIF) [file pgen.1004148.s008.tif]

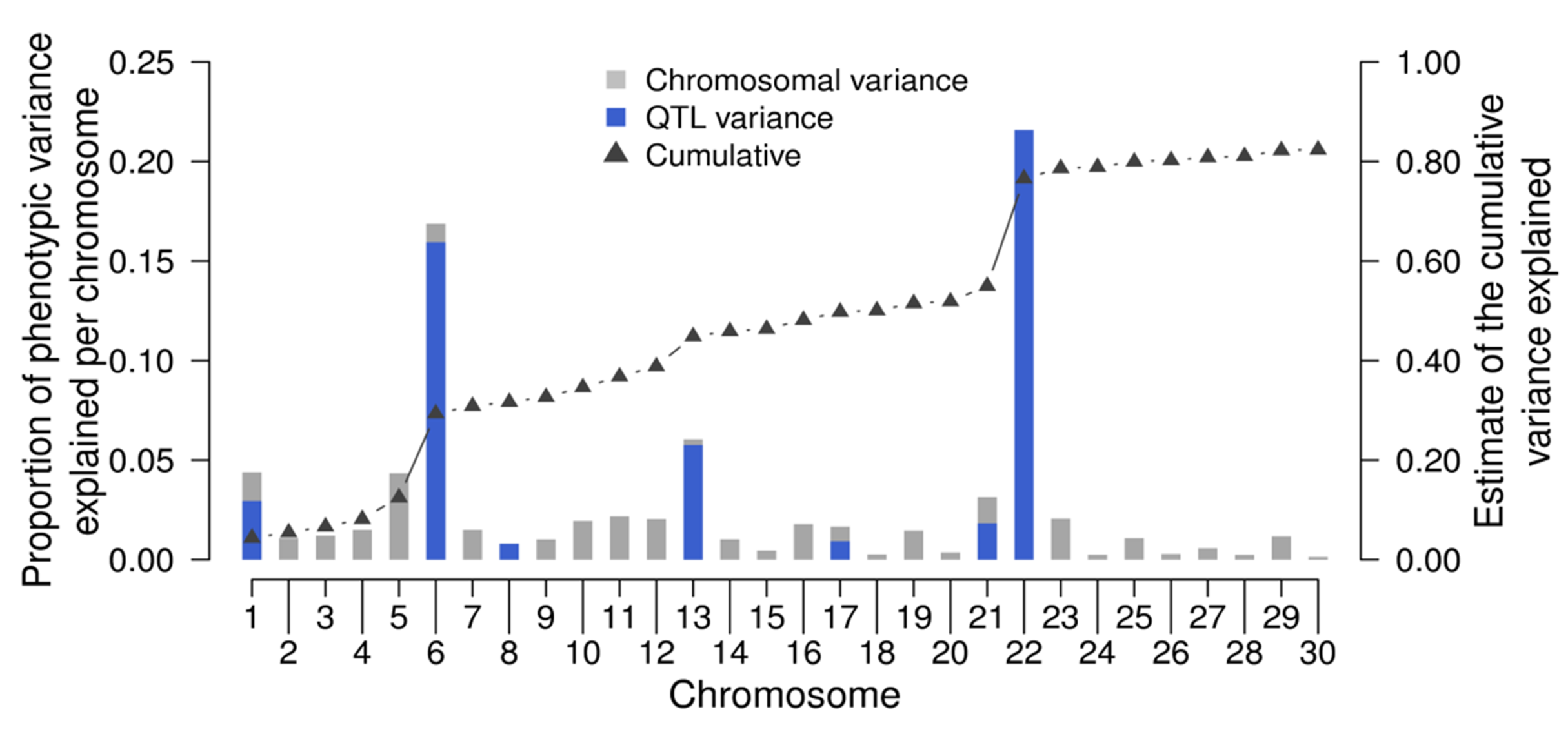

Supplement: Figure S9 — Chromosomal partitioning of the phenotypic variance of the ‘proportion of daughters without spotting’. The grey and blue bars indicate the fraction of phenotypic variance attributed to a particular chromosome and QTL region, respectively. The triangles represent the cumulative proportion of phenotypic variance attributable to the 30 chromosomes. (TIF) [file pgen.1004148.s009.tif]

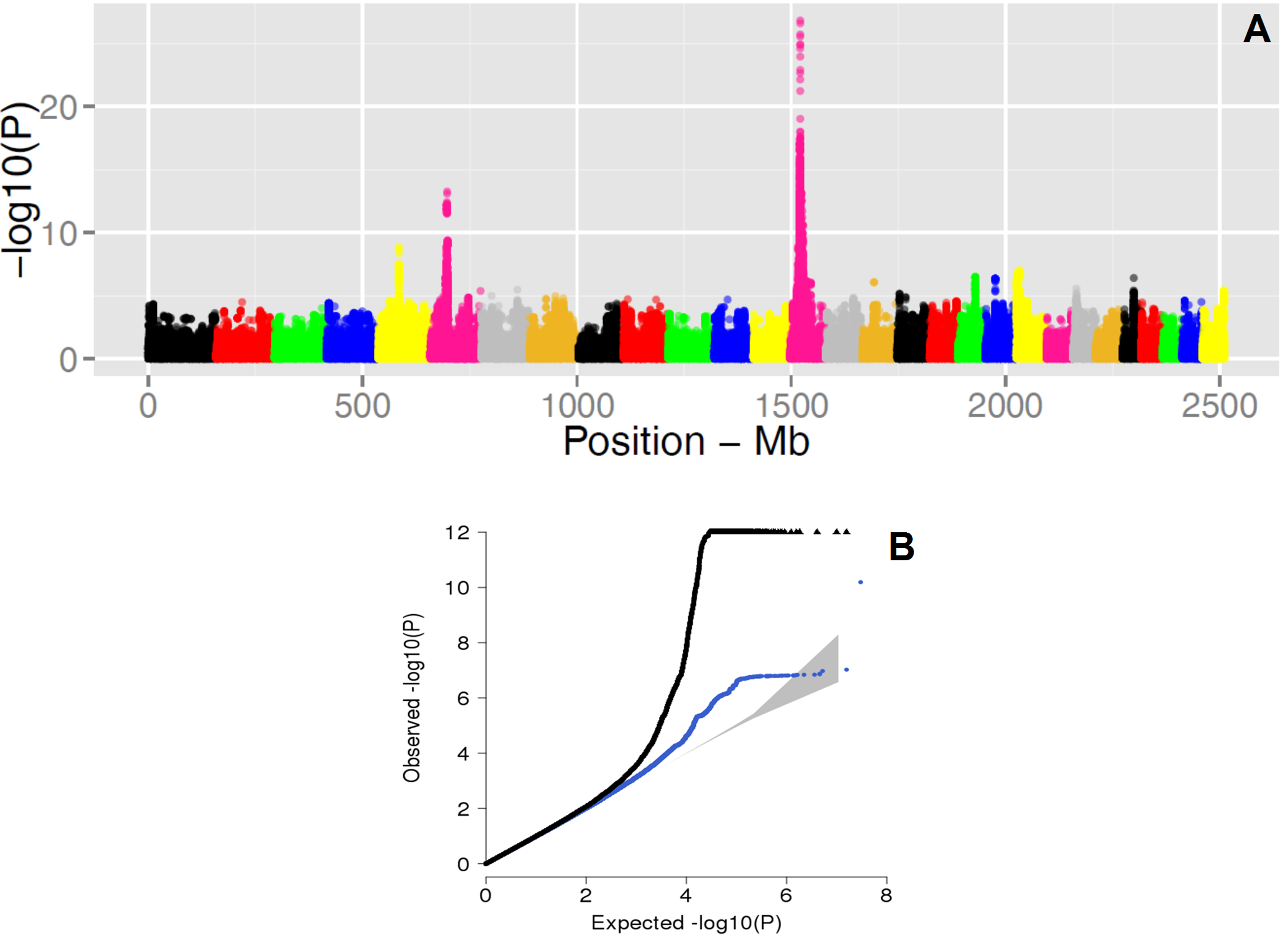

Supplement: Figure S10 — Manhattan plot of association of 15'182'131 imputed variants with the body size in 3602 Fleckvieh animals (A). Panel B represents corresponding quantile-quantile plot. Shown in blue is the quantile-quantile plot resulting from excluding SNPs in the region of significant genes. The shaded area represents 95% concentration band under the null hypothesis of no association. (TIF) [file pgen.1004148.s010.tif]

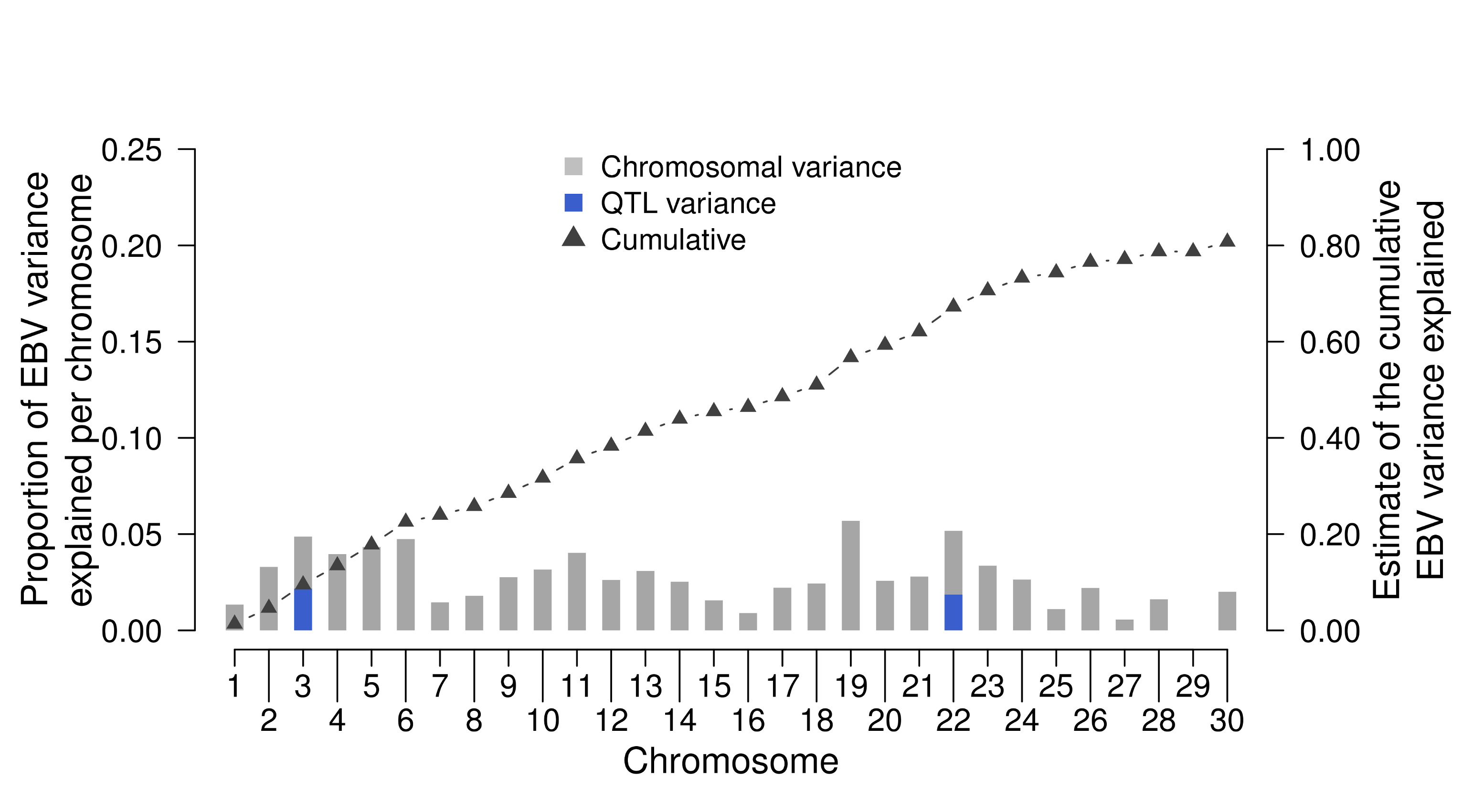

Supplement: Figure S11 — Chromosomal partitioning of the EBV variance of the ‘somatic cell count’ trait. The grey and blue bars indicate the fraction of phenotypic variance attributed to a particular chromosome and QTL region, respectively. The triangles represent the cumulative proportion of phenotypic variance attributable to the 30 chromosomes. (TIF) [file pgen.1004148.s011.tif]

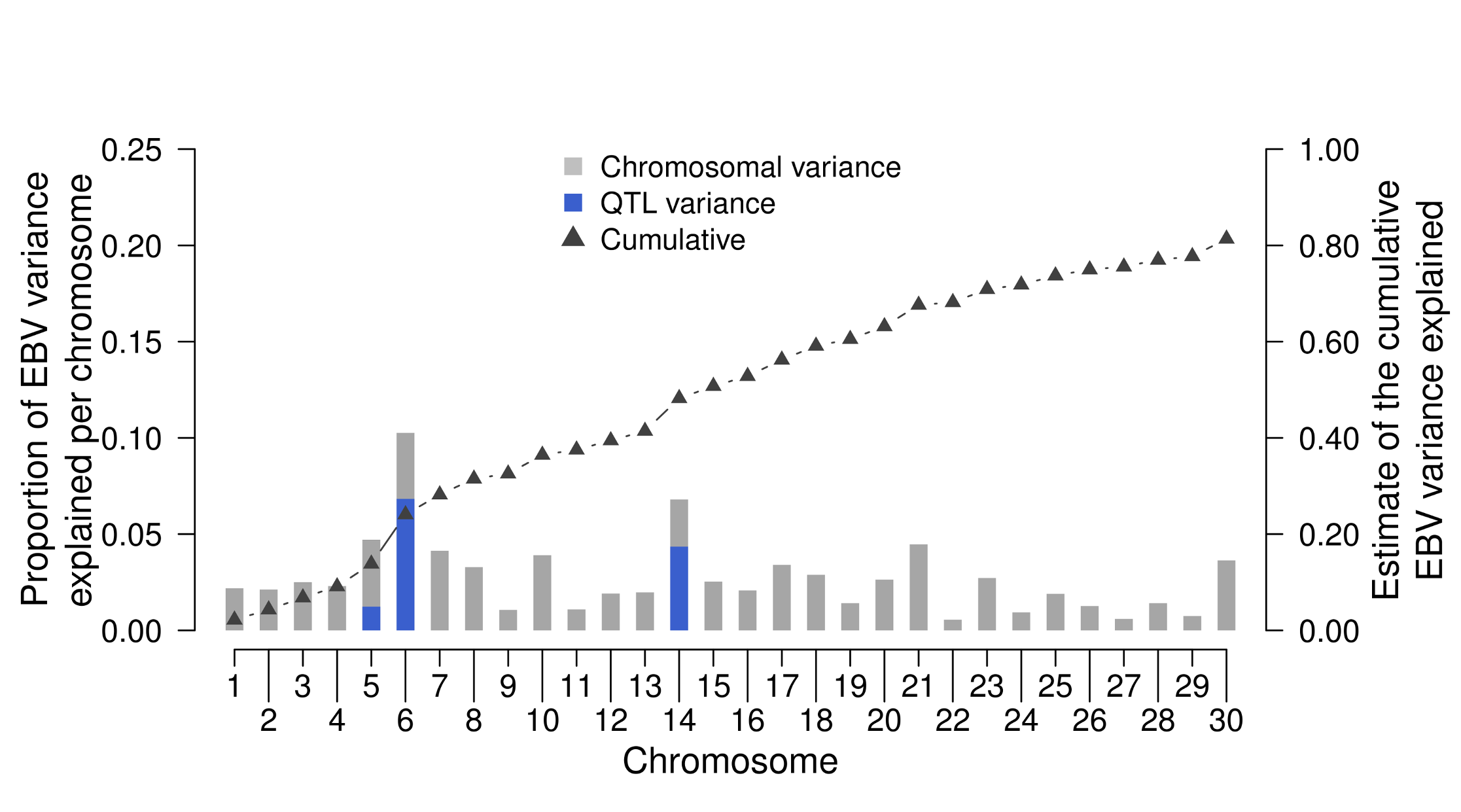

Supplement: Figure S12 — Chromosomal partitioning of the EBV variance of the ‘body size’ trait. The grey and blue bars indicate the fraction of phenotypic variance attributed to a particular chromosome and QTL region, respectively. The triangles represent the cumulative proportion of phenotypic variance attributable to the 30 chromosomes. (TIF) [file pgen.1004148.s012.tif]

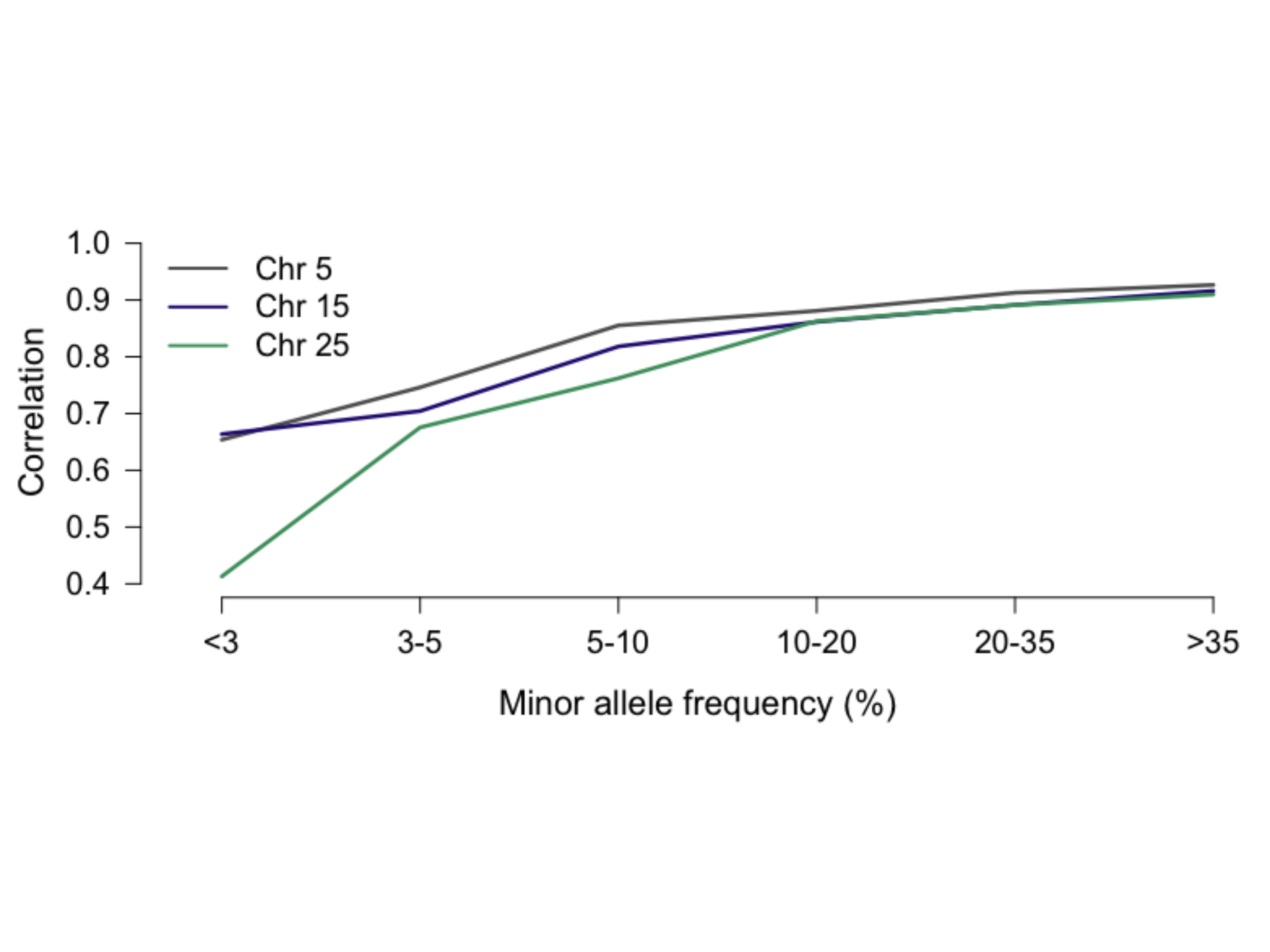

Supplement: Figure S13 — Evaluation of imputation accuracy. Correlation between imputed and array-derived genotypes as a function of the minor allele frequency. Genotypes for randomly selected SNPs were set to missing and subsequently imputed based on sequence-derived genotypes of 43 re-sequenced animals. (TIF) [file pgen.1004148.s013.tif]

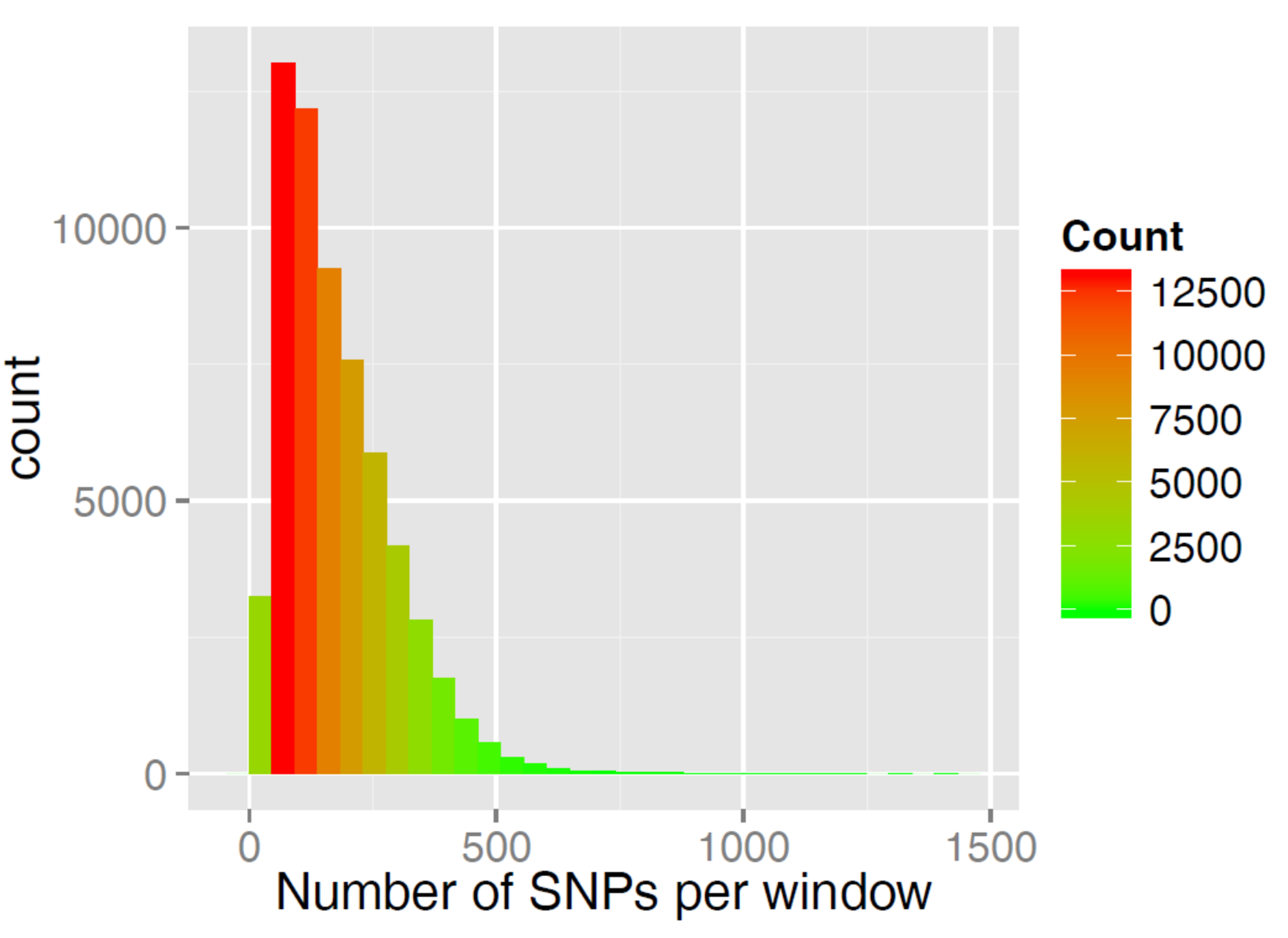

Supplement: Figure S14 — Distribution of the number of SNPs in 62'196 non-overlapping windows along BTA1 to BTA29. (TIF) [file pgen.1004148.s014.tif]
