# Supplementary material for: Classic Selective Sweeps Revealed by Massive Sequencing in Cattle
Source: PLoS Genet. 2014 Feb 27;10(2):e1004148. doi: 10.1371/journal.pgen.1004148 (PMC3937232; doi:10.1371/journal.pgen.1004148)
Supplement: Table S1 — Comparison of fraction (%) of marker pairs with different r2 levels as a function of distance (kb). (DOCX) [file pgen.1004148.s015.docx]

| **Table S1. Comparison of fraction (%) of marker pairs with different *r^2^* levels as a function of distance (kb).** | | | | | | | | | | | | |
| --- | --- | --- | --- | --- | --- | --- | --- | --- | --- | --- | --- | --- |
|  | **Distance bin** | | | | | | | | | | | |
| ***r^2^*** | **<2** | **2-3.5** | **3.5-5** | **5-10** | **10-20** | **20-30** | **30-40** | **40-50** | **50-100** | **100-250** | **250-500** | **500-1000** |
| **0.1-0.25** | 0.231 | 0.218 | 0.247 | 0.294 | 0.373 | 0.444 | 0.573 | 0.621 | 0.716 | 0.830 | 0.932 | 0.948 |
| **0.25-0.4** | 0.145 | 0.129 | 0.138 | 0.186 | 0.211 | 0.241 | 0.210 | 0.190 | 0.166 | 0.119 | 0.053 | 0.046 |
| **0.4-0.6** | 0.137 | 0.196 | 0.191 | 0.183 | 0.204 | 0.184 | 0.141 | 0.131 | 0.090 | 0.042 | 0.012 | 0.004 |
| **0.6-1** | 0.486 | 0.457 | 0.425 | 0.337 | 0.212 | 0.131 | 0.076 | 0.059 | 0.028 | 0.009 | 0.002 | 0.002 |

LD is estimated for marker pairs in different distance bins as <2, 2–3. 5, 3.5–5, 5–10, 10–20, 20–30, 30–40, 40–50, 50-100, 100-250, 250-500, 500-1000 kilobasepairs.
