# Supplementary material for: Classic Selective Sweeps Revealed by Massive Sequencing in Cattle
Source: PLoS Genet. 2014 Feb 27;10(2):e1004148. doi: 10.1371/journal.pgen.1004148 (PMC3937232; doi:10.1371/journal.pgen.1004148)
Supplement: Table S2 — Summary statistics of testing the difference between sequence vs., array based LD. (DOCX) [file pgen.1004148.s016.docx]

| **Table S2. Summary statistics of testing the difference between sequence vs., array based LD.** | | | | | | | |
| --- | --- | --- | --- | --- | --- | --- | --- |
| **Distance**  **bin (kb)** | **^1^Seq**  **(*r^2^*)** | **Seq**  **(sd)** | **^2^Seq**  **(# pair)** | **^1^Array**  **(*r^2^*)** | **Array**  **(sd)** | **^2^Array**  **(# pair)** | **t-test statistic** |
| <2 | 0.35 | 0.36 | 14,485 | 0.57 | 0.33 | 2816 | 39.14*** |
| 2 - 3.5 | 0.27 | 0.32 | 11,710 | 0.50 | 0.33 | 3,031 | 44.44*** |
| 3.5 - 5 | 0.23 | 0.30 | 11,291 | 0.44 | 0.32 | 3,136 | 43.50*** |
| 5 - 10 | 0.18 | 0.26 | 36,106 | 0.37 | 0.31 | 9,816 | 69.47*** |
| 10 - 20 | 0.11 | 0.20 | 67,817 | 0.27 | 0.27 | 18,529 | 82.73*** |
| 20 - 30 | 0.08 | 0.16 | 65,222 | 0.18 | 0.22 | 17,761 | 63.92*** |
| 30 - 50 | 0.06 | 0.13 | 63,421 | 0.13 | 0.18 | 17,555 | 50.90*** |
| 50 - 70 | 0.05 | 0.11 | 62,107 | 0.10 | 0.15 | 17,282 | 44.57*** |
| 70 - 100 | 0.04 | 0.08 | 292,605 | 0.06 | 0.10 | 83,912 | 72.85*** |
| 100 - 250 | 0.02 | 0.06 | 835,312 | 0.03 | 0.04 | 243,628 | 82.59*** |
| 250 - 500 | 0.02 | 0.04 | 1,383,490 | 0.02 | 0.03 | 410,209 | 76.33*** |

^1^ mean LD estimated in a corresponding distance bin.

^2^ the number of pairwise estimates of LD in each bin.
