# Supplementary material for: Classic Selective Sweeps Revealed by Massive Sequencing in Cattle
Source: PLoS Genet. 2014 Feb 27;10(2):e1004148. doi: 10.1371/journal.pgen.1004148 (PMC3937232; doi:10.1371/journal.pgen.1004148)
Supplement: Table S3 — Candidate regions identified by iHS analysis. (DOCX) [file pgen.1004148.s017.docx]

| **Table S3. Candidate regions identified by iHS analysis.** | | | | | | | |
| --- | --- | --- | --- | --- | --- | --- | --- |
| **#** | **Chr** | **Position-bp** | **\|iHS\|** | **P-value** | **Gene** | **Function/Association** | **Reference** |
| 1 | 6 | 72171128 | 2.82 | 0.00029 | *KIT* | Pigmentation |  |
| 2 | 1 | 1915043 | 2.81 | 0.00031 | *OLIG1* |  |  |
| 3 | 14 | 42856951 | 2.79 | 0.00034 | *LOC100335199* | Tescalcin-like with unknown function |  |
| 4 | 24 | 31312327 | 2.76 | 0.00039 | *ZNF521* | Zinc finger protein 521 |  |
| 5 | 4 | 80476509 | 2.69 | 0.00047 | *Gene desert* | Gene desert |  |
| 6 | 23 | 13483561 | 2.67 | 0.00051 | *KIF6-like* | Kinesin-like protein KIF6-like |  |
| 7 | 11 | 98618477 | 2.66 | 0.00053 | *SPTAN1* | Early Infantile Epileptic Syndrome |  |
| 8 | 14 | 42271706 | 2.64 | 0.00056 | *MAGEA13P-like* | Immune system |  |
| 9 | 8 | 1.08E+08 | 2.64 | 0.00058 | *ASTN2* | Astrotactin 2 |  |
| 10 | 3 | 78587030 | 2.63 | 0.00061 | *SLC35D1* | Responsible for skeletal dysplasia | Hiraoka et al. 2007 |
| 11 | 2 | 49274059 | 2.62 | 0.00068 | *SNRPD1* |  |  |
| 12 | 7 | 60389472 | 2.59 | 0.00074 | *Gene desert* | Gene desert |  |
| 13 | 3 | 1.09E+08 | 2.58 | 0.00076 | *GRIK3* | Reward-related learning | Minelli et al. 2009 |
| 14 | 5 | 68743174 | 2.58 | 0.00077 | *SLC41A2* |  |  |
| 15 | 15 | 68944767 | 2.58 | 0.00079 | *Gene desert* | Gene desert |  |
| 16 | 20 | 21281507 | 2.57 | 0.00082 | *TAOK1* |  |  |
| 17 | 9 | 75891505 | 2.56 | 0.00085 | *PEX7* | Peroxisomal biogenesis factor 7_Peroxisome biogenesis disorders__nerveus |  |
| 18 | 17 | 48809343 | 2.56 | 0.00087 | *TMEM132D* | Panic disorder | Erhardt et al. 2012 |
| 19 | 4 | 52237367 | 2.55 | 0.00092 | *CAV1 & CAV2* | Cystic Fibrosis |  |
| 20 | 7 | 59299557 | 2.53 | 0.00093 | *PLAC8L1* | Placenta like 8 |  |
| 21 | 18 | 36175506 | 2.53 | 0.00095 | *CHD1* | Cadherin1 |  |
| 22 | 2 | 71544511 | 2.48 | 0.00105 | *DBI* | Diazepam receptor- acyl-CoA binding Protein |  |
| 23 | 7 | 43810382 | 2.45 | 0.00113 | *ORF* | Olfactory receptor family cluster 2 |  |
| 24 | 20 | 3808800 | 2.44 | 0.00119 | *STK10* |  |  |
| 25 | 19 | 52600264 | 2.43 | 0.00121 | *NPTX1* | Neuronal pentraxin I, exclusively localized to the nervous system |  |
| 26 | 16 | 46579025 | 2.4 | 0.00132 | *TNFRSF9* | Tumor necrosis factor receptor superfamily_Imune system |  |
| 27 | 3 | 13339357 | 2.39 | 0.00141 | *FCRL4* | Immunoglobulin |  |
| 28 | 4 | 88279974 | 2.39 | 0.00143 | *TAS2R16* | Bitter taste receptor, type 2 |  |
| 29 | 2 | 12288594 | 2.36 | 0.0015 | *Gene desert* | Gene desert |  |
| 30 | 9 | 43460434 | 2.36 | 0.00153 | *MGC12345* | Hypothetical protein |  |
| 31 | 22 | 32017564 | 2.32 | 0.00172 | *MITF* | Pigmentation |  |
| 32 | 4 | 54485270 | 2.31 | 0.00183 | *SETMAR gene* | SETMAR gene retinal pigmentation |  |
| 33 | 21 | 24063814 | 2.29 | 0.00196 | *NDUFB1* | NADH dehydrogenase [ubiquinone] 1 beta subcomplex subunit 1 pseudogene |  |
| 34 | 2 | 99904366 | 2.27 | 0.00203 | *ERBB4* | Pigmentation/ Neurobehavioral functioning | Pickrell et al. 2009 |
| 35 | 25 | 25879529 | 2.26 | 0.00206 | *XPO6* | XPO6 |  |
| 36 | 9 | 1.04E+08 | 2.24 | 0.0022 | *UNC93A* | unc-93 homolog A_linked to TLR and associated with herpes simplex encephalitis type 1 |  |
| 37 | 11 | 1.01E+08 | 2.24 | 0.00222 | *LAMC3* | Cause malformations of occipital cortical development | Barak et al. 2011 |
| 38 | 10 | 43656593 | 2.23 | 0.00227 | *ATL1* | formation and growth of axons |  |
| 39 | 1 | 1.42E+08 | 2.15 | 0.00281 | *DSCAM* | Down syndrome cell adhesion molecule |  |
| 40 | 15 | 31196812 | 2.14 | 0.00294 | *Gene desert* | Gene desert |  |
| 41 | 21 | 32012049 | 2.07 | 0.00368 | *NRG4* | Pigmentation/ Neurobehavioral functioning | Pickrell et al. 2009 |
| 42 | 7 | 95960458 | 2.06 | 0.00387 | *FAM172A* | conserved genes_FAM172A family with sequence similarity 172 member A |  |
| 43 | 14 | 58196348 | 2.05 | 0.00389 | *EMC2* | Candidate gene for developmental dyslexia |  |
| 44 | 11 | 33156535 | 2.05 | 0.00396 | *NRXN1* | Nervous system |  |
| 45 | 26 | 46698960 | 2.03 | 0.00415 | *Gene desert* | Gene desert |  |
| 46 | 4 | 1.11E+08 | 2.02 | 0.00437 | *Cullin1* | Cullin1 |  |
| 47 | 23 | 39053772 | 2.01 | 0.00449 | *RNF144B* | ring finger protein 144B |  |
| 48 | 27 | 34339543 | 1.98 | 0.0049 | *ADAM2* | ADAM metallopeptidase domain 2 |  |
| 49 | 25 | 23942521 | 1.97 | 0.00494 | *HS3ST4* | Involved in blood clotting |  |
| 50 | 7 | 15246693 | 1.97 | 0.00502 | *ORF* | Olfactory receptor family cluster |  |
| 51 | 4 | 3739008 | 1.95 | 0.00508 | *Gene desert* | Gene desert |  |
| 52 | 6 | 29274371 | 1.87 | 0.00637 | *Gene desert* | Gene desert |  |
| 53 | 28 | 4020722 | 1.86 | 0.00669 | *Pol (HIV)* | Pol (HIV) like gene |  |
| 54 | 17 | 20477583 | 1.85 | 0.00677 | *PCDH18* | Protocadherin 18 |  |
| 55 | 22 | 55713616 | 1.85 | 0.00688 | *SLC6A1* | Solute carrier family 6 (neurotransmitter transporter, GABA), member 1_nervus system |  |
| 56 | 17 | 40044391 | 1.85 | 0.00691 | *ULBP3* | “Sudden whitening of the hair” phenomenon | Petukhova et al. 2010 |
| 57 | 14 | 36928094 | 1.84 | 0.00695 | *EYA1* | Eyes absent homolog 1, Eye abnormalities and branchiootorenal syndrome |  |
| 58 | 11 | 1.04E+08 | 1.84 | 0.00711 | *ADAMTS13* | involved in blood clotting |  |
| 59 | 2 | 56501561 | 1.81 | 0.00777 | *LRP1B* | Low density lipoprotein receptor-related protein 1B |  |
| 60 | 18 | 14826364 | 1.8 | 0.00785 | *MC1R* | Pigmentation |  |
| 61 | 14 | 23922721 | 1.8 | 0.00789 | *LOC100847881* | uncharacterized LOC100847881 |  |
| 62 | 1 | 56375092 | 1.78 | 0.00862 | *PVRL3* | Poliovirus receptor-related 3-like |  |
| 63 | 10 | 97286694 | 1.77 | 0.00878 | *MIR2293* | Regulation of stress signaling pathways_noncoding regions |  |
| 64 | 12 | 17596335 | 1.77 | 0.00888 | *Gene desert* | Gene desert |  |
| 65 | 4 | 20025093 | 1.76 | 0.00918 | *VWDE* | Involved in blood clotting |  |
| 66 | 25 | 42035012 | 1.75 | 0.00944 | *MAFK* | v-maf musculoaponeurotic fibrosarcoma oncogene homolog K (avian) |  |
| 67 | 9 | 45672801 | 1.74 | 0.00982 | *LIN28B* | lin-28 homolog B_enhances the translation of the IGF-2_cause gigantism and a delay in puberty |  |
