# Supplementary material for: Classic Selective Sweeps Revealed by Massive Sequencing in Cattle
Source: PLoS Genet. 2014 Feb 27;10(2):e1004148. doi: 10.1371/journal.pgen.1004148 (PMC3937232; doi:10.1371/journal.pgen.1004148)
Supplement: Table S4 — Candidate regions identified by CLR analysis. (DOCX) [file pgen.1004148.s018.docx]

| **Table S4. Candidate regions identified by CLR analysis.** | | | | | | | |
| --- | --- | --- | --- | --- | --- | --- | --- |
| # | **Chr** | **Position-bp** | **CLR** | **P-value** | **Gene** | **Function/Association** | **Reference** |
| 1 | 5 | 117550201 | 693.8 | 0.00002 |  | Gene desert |  |
| 2 | 1 | 56460462 | 436.4 | 0.00003 | *PVRL3* | Poliovirus receptor-related 3-like |  |
| 3 | 1 | 141931077 | 327.6 | 0.00005 | *DSCAM* | Down syndrome cell adhesion molecule |  |
| 4 | 14 | 42824451 | 260.8 | 0.00010 | *LOC100335199* | Tescalcin-like with unknown function |  |
| 5 | 6 | 71395199 | 247.7 | 0.00011 | *KIT* | Pigmentation |  |
| 6 | 5 | 109068036 | 245.1 | 0.00013 | *CACNA1C* | Bipolar disorder and schizophrenia | Nyegaard et al. 2010 |
| 7 | 1 | 2401098 | 207.9 | 0.00016 | *OLIG1* | OLIG1 |  |
| 8 | 21 | 21554828 | 206.5 | 0.00018 | *NDUFB1* | NADH dehydrogenase [ubiquinone] 1 beta subcomplex subunit 1 pseudogene |  |
| 9 | 17 | 48687451 | 191.5 | 0.00021 | *TMEM132D* | Panic disorder | Erhardt et al. 2012 |
| 10 | 28 | 4006967 | 180.9 | 0.00022 | *Pol like* | Pol (HIV) like gene |  |
| 11 | 27 | 36837623 | 161.2 | 0.00029 | *Ank-1* | Hemolytic anaemia disorder | Gallagher et al. 1997 |
| 12 | 25 | 40219849 | 159.8 | 0.00030 | *MAFK* | v-maf musculoaponeurotic fibrosarcoma oncogene homolog K (avian) |  |
| 13 | 12 | 46021767 | 139.1 | 0.00033 |  | Gene desert |  |
| 14 | 2 | 61026902 | 125.1 | 0.00037 |  | Gene desert |  |
| 15 | 2 | 73352329 | 108.7 | 0.00038 | *TFCP2L1* | transcription factor CP2-like 1 |  |
| 16 | 1 | 35212851 | 104.1 | 0.00041 |  | Gene desert |  |
| 17 | 8 | 96134318 | 103.7 | 0.00043 | *OR13C8* | olfactory receptor, family 13, subfamily C, member 8 |  |
| 18 | 15 | 50749853 | 96.1 | 0.00048 | *OR51A7* | olfactory receptor, family 51, subfamily A, member 7 |  |
| 19 | 1 | 26489726 | 94.3 | 0.00049 | *ROBO1* | Regulates Interaural Interaction in Auditory Pathways | Lamminmäki et a. 2012 |
| 20 | 20 | 3802285 | 90.7 | 0.00051 | *STK10* |  |  |
| 21 | 1 | 13485068 | 89.3 | 0.00054 |  | Gene desert |  |
| 22 | 1 | 30211059 | 81.5 | 0.00061 | *LOC100337296* | Leukocyte immunoglobulin-like receptor, subfamily A (with TM domain), member 6-like |  |
| 23 | 1 | 124644885 | 74.0 | 0.00067 |  | Gene desert |  |
| 24 | 20 | 21252540 | 66.3 | 0.00086 | *TAOK1* | TAOK1 |  |
| 25 | 10 | 10487027 | 62.7 | 0.00091 |  | Gene desert |  |
| 26 | 5 | 44291497 | 61.9 | 0.00099 | *LOC100138933* | E3 ubiquitin-protein ligase MARCH6-like |  |
| 27 | 4 | 17369825 | 61.2 | 0.00104 | *LOC614650* | Elongation factor 1-beta-like |  |
| 28 | 8 | 33180418 | 59.6 | 0.00111 |  | Gene desert |  |
| 29 | 10 | 23254876 | 55.7 | 0.00121 | *LOC100296317* | T-cell receptor alpha chain V region HPB-MLT-like |  |
| 30 | 27 | 34355126 | 54.6 | 0.00124 | *ADAM2* | ADAM metallopeptidase domain 2 |  |
| 31 | 18 | 36144400 | 51.7 | 0.00134 | *CDH1* | Cadherin1 |  |
| 32 | 21 | 44609230 | 51.2 | 0.00137 | *NPAS3* | High risk of developing schizophrenia | Yu et al. 2013 |
| 33 | 23 | 13453357 | 48.5 | 0.00151 | *KIF6-like* | kinesin-like protein KIF6-like |  |
| 34 | 18 | 14930982 | 47.5 | 0.00156 | *MC1R* | Pigmentation |  |
| 35 | 9 | 45630508 | 43.8 | 0.00166 | *LIN28B* | cause gigantism and a delay in puberty | Zhu et al. 2010 |
| 36 | 26 | 46556687 | 43.4 | 0.00169 |  | Gene desert |  |
| 37 | 1 | 19647275 | 42.7 | 0.00175 |  | Gene desert |  |
| 38 | 14 | 42224109 | 42.6 | 0.00178 | *MAGEA13PL* | immune system |  |
| 39 | 2 | 12205403 | 41.1 | 0.00185 |  | Gene desert |  |
| 40 | 11 | 99102317 | 40.8 | 0.00186 | *SPTAN1* | spectrin, alpha, non-erythrocytic 1_Early Infantile Epileptic Syndrome |  |
| 41 | 9 | 75889472 | 40.2 | 0.00188 | *PEX7* | peroxisomal biogenesis factor 7_Peroxisome biogenesis disorders__nerveus |  |
| 42 | 7 | 15331252 | 38.8 | 0.00194 | *ORF* | olfactory receptor family cluster |  |
| 43 | 16 | 45905057 | 35.7 | 0.00207 | *TNFRSF9* | tumor necrosis factor receptor superfamily_Imune system |  |
| 44 | 21 | 32035358 | 34.8 | 0.00221 | *NRG4* | Pigmentation/ Neurobehavioral functioning | Pickrell et al. 2009 |
| 45 | 7 | 58881309 | 34.4 | 0.00226 | *PLAC8L1* | Placenta like 8 |  |
| 46 | 7 | 38787441 | 30.8 | 0.00256 | *SEMA6A* | sema domain, transmembrane domain (TM), and cytoplasmic domain, (semaphorin) 6A |  |
| 47 | 8 | 108060740 | 28.9 | 0.00269 | *ASTN2* | Astrotactin 2 |  |
| 48 | 4 | 4522805 | 27.3 | 0.00293 | *COBL* | Cordon-bleu homolog (mouse) |  |
| 49 | 3 | 17810479 | 25.7 | 0.00317 | *FCRL4* | Immunoglobulin |  |
| 50 | 11 | 101183624 | 25.0 | 0.00325 | *LAMC3* | Cause malformations of occipital cortical development | Barak et al. 2011 |
| 51 | 9 | 43469153 | 23.9 | 0.00355 | *MGC12345* | hypotecktocal protein |  |
| 52 | 27 | 18018699 | 23.6 | 0.00365 | *LOC781220* | Chromobox homolog 1 (HP1 beta homolog Drosophila ) |  |
| 53 | 24 | 31597845 | 23.2 | 0.00384 | *ZNF521* | Zinc finger protein 521 |  |
| 54 | 14 | 24053745 | 19.2 | 0.00476 | *LOC100847881* | uncharacterized |  |
| 55 | 25 | 30325134 | 17.9 | 0.00532 | *XPO6* | Exportin-6 |  |
| 56 | 21 | 10451546 | 17.6 | 0.00538 | *NR2F2* | Development of a number of tissues and organs including heart, blood vessels, muscles and limbs. |  |
| 57 | 7 | 36906142 | 17.4 | 0.00545 |  | Gene desert |  |
| 58 | 4 | 54390052 | 17.4 | 0.00546 | *SETMAR* | Retinal pigmentation |  |
| 59 | 10 | 43787497 | 17.2 | 0.00557 | *ATL1* | Formation and growth of axons |  |
| 60 | 7 | 52837137 | 16.4 | 0.00589 | *LOC783452* | Pro-neuregulin-2, membrane-bound isoform-like |  |
| 61 | 12 | 70473320 | 16.1 | 0.00602 | *LOC100336951* | Multidrug resistance-associated protein 4-like |  |
| 62 | 22 | 31640424 | 14.0 | 0.00709 | *MITF* | Pigmentation |  |
| 63 | 3 | 57433341 | 14.0 | 0.00710 | *CLCA3* | Chloride channel, calcium activated, family member 3 |  |
| 64 | 10 | 97740663 | 13.9 | 0.00718 | *MIR2293* | Regulation of stress signaling pathways |  |
| 65 | 11 | 104345611 | 13.5 | 0.00736 | *ADAMTS13* | Involved in blood clotting |  |
| 66 | 3 | 109583431 | 13.1 | 0.00761 | *GRIK3* | Reward-related learning | Minelli et al. 2009 |
| 67 | 17 | 20459946 | 12.7 | 0.00785 | *PCDH18* | Protocadherin 18 |  |
| 68 | 3 | 78685603 | 12.0 | 0.00854 | *SLC35D1* | Responsible for skeletal dysplasia | Hiraoka et al. 2007 |
| 69 | 2 | 100084101 | 11.9 | 0.00855 | *ERBB4* | Pigmentation/ Neurobehavioral functioning | Pickrell et al. 2009 |
| 70 | 4 | 88368618 | 11.7 | 0.00887 | *TAS2R16* | Bitter taste receptor, type 2 |  |
| 71 | 21 | 13294682 | 11.6 | 0.00897 | *LOC617694* | NADH dehydrogenase (ubiquinone) 1 beta subcomplex, 4, 15kDa pseudogene |  |
| 72 | 7 | 43710839 | 11.5 | 0.00905 | *ORF* | Olfactory receptor family cluster 2 |  |
| 73 | 17 | 39678673 | 11.4 | 0.00917 | *ULBP3* | “Sudden whitening of the hair” phenomenon | Petukhova et al. 2010 |
