# Supplementary material for: Classic Selective Sweeps Revealed by Massive Sequencing in Cattle
Source: PLoS Genet. 2014 Feb 27;10(2):e1004148. doi: 10.1371/journal.pgen.1004148 (PMC3937232; doi:10.1371/journal.pgen.1004148)
Supplement: Table S5 — Results of GWA studies based on estimated breeding values for Somatic cell count and body size in Fleckvieh animals. (DOCX) [file pgen.1004148.s019.docx]

Table S5. Results of GWA studies based on estimated breeding values for Somatic cell count and body size in Fleckvieh animals.

| Chr | Position (bp) | Top-SNP (NCBI reference ID) | Minor allele frequency | P-Value | Candidate gene | Proportion of EBV variance explained [%] |
| --- | --- | --- | --- | --- | --- | --- |
| **Somatic cell count** | |  |  |  |  |  |
| 3 | 15,613,949 | rs386094483 | 0.13 | 7.9 x 10^-11^ | *DCST1* | 2.17% |
| 22 | 53,514,171 | rs109291016 | 0.43 | 6.1 x 10^-11^ | *LTF* | 1.86% |
| **Body size** | |  |  |  |  |  |
| 5 | 47,999,663 | rs378550908 | 0.04 | 1.4 x 10^-9^ | *LOC100297155* | 1.23% |
| 6 | 39,103,880 | rs211652883 | 0.01 | 5.4 x 10^-14^ | *NCAPG* | 6.83% |
| 14 | 25,015,640 | rs109815800 | 0.12 | 1.5 x 10^-27^ | *PLAG1* | 4.35% |
